# Climate change and impacts in the Eastern Mediterranean and the Middle East

**DOI:** 10.1007/s10584-012-0418-4

**Published:** 2012-03-07

**Authors:** J. Lelieveld, P. Hadjinicolaou, E. Kostopoulou, J. Chenoweth, M. El Maayar, C. Giannakopoulos, C. Hannides, M. A. Lange, M. Tanarhte, E. Tyrlis, E. Xoplaki

**Affiliations:** 1 The Cyprus Institute, P.O. Box 27456, 1645 Nicosia, Cyprus; 2 Max Planck Institute for Chemistry, 55020 Mainz, Germany; 3 King Saud University, Riyadh, 11451 Saudi Arabia; 4 University of Surrey, Guildford, Surrey UK GU2 7XH; 5 National Observatory of Athens, 15236 Athens, Greece; 6 University of the Aegean, 81100 Mytilene, Greece; 7 Justus-Liebig University of Giessen, 35390 Giessen, Germany; 8 University of Bern, 3012 Bern, Switzerland

## Abstract

The Eastern Mediterranean and the Middle East (EMME) are likely to be greatly affected by climate change, associated with increases in the frequency and intensity of droughts and hot weather conditions. Since the region is diverse and extreme climate conditions already common, the impacts will be disproportional. We have analyzed long-term meteorological datasets along with regional climate model projections for the 21st century, based on the intermediate IPCC SRES scenario A1B. This suggests a continual, gradual and relatively strong warming of about 3.5–7°C between the 1961–1990 reference period and the period 2070–2099. Daytime maximum temperatures appear to increase most rapidly in the northern part of the region, i.e. the Balkan Peninsula and Turkey. Hot summer conditions that rarely occurred in the reference period may become the norm by the middle and the end of the 21st century. Projected precipitation changes are quite variable. Annual precipitation is expected to decrease in the southern Europe – Turkey region and the Levant, whereas in the Arabian Gulf area it may increase. In the former region rainfall is actually expected to increase in winter, while decreasing in spring and summer, with a substantial increase of the number of days without rainfall. Anticipated regional impacts of climate change include heat stress, associated with poor air quality in the urban environment, and increasing scarcity of fresh water in the Levant.

**Electronic supplementary material:**

The online version of this article (doi:10.1007/s10584-012-0418-4) contains supplementary material, which is available to authorized users.

## Introduction

The climate of the northern EMME is mostly temperate with warm to hot, dry summers, occasional droughts, and mild, relatively wet winters (Bolle 2003; Lionello et al. 2006). In the southern EMME, an arid hot desert climate prevails, where precipitation is low and vegetation scanty (Issar and Zohar 2007). The temperature and precipitation gradients across the region are remarkable. In some areas precipitation is among the highest in Europe, e.g. up to 2,000 mm/year or more over the mountains of the Dinaric Alps, the Caucasus and the Zagros mountains, while several degrees latitude further south this can be orders of magnitude less. The precipitation is most abundant in winter and largely associated with the southward propagation of the polar front jet, which directs cyclonic disturbances over the region (Krichak et al. 2000).

Precipitation patterns in the EMME do not only depend upon the synoptic weather conditions but also on the pronounced topography, for example the Taurus and Zagros Mountains through which the Euphrates and Tigris rivers supply the much needed water downstream (Barth and Steinkohl 2004; Evans et al. 2004). Climate change projections suggest a northward advancement of the jet stream and the storm track in the 21st century, which may increase the regional dependency on orographic rainfall (Black et al. 2010; Evans 2010). Precipitation could potentially increase in mountainous regions in the northern EMME, and possibly also in the southern EMME owing to the northward expansion of the tropical rain belt (Önol and Semazzi 2009; Evans 2010).

Nonetheless, climate models consistently predict an overall drying of the region (Gibelin and Déqué 2003; IPCC 2007; Kitoh et al. 2008), which will impact major river systems and the downstream water resources and food production. The area of rainfed agricultural land may decrease, and prolongation of the dry season will reduce the availability of rangelands for grazing animals (Evans 2009). Although crop yields could potentially increase in winter, they may decrease during the rest of the year, and the increased risk of vegetation fires and air pollution can aggravate the environmental stress (Giannakopoulos et al. 2009).

Gradients and contrasts are characteristic for the EMME, not only in climatic conditions, but also in social and economic aspects, access to natural resources, as well as cultural and religious traditions. This diversity is a regional attribute, but can also be associated with political tensions. Since the region is a primary climate change “hot spot”, there is concern about the future state of the environment and societal consequences (Giorgi 2006; IPCC 2007). A large part of the EMME is notorious for water scarcity. The demand for fresh water increases continually, related to population growth and economic development, and the Middle East has been identified as the first region worldwide to effectively run out of fresh water (Allan 2001). Although particular sub-regions are unsuitable for agriculture, others are highly dependent on it and climate change is likely to impose challenges that could exacerbate regional tensions. In summer the EMME can be hot, and climate change may intensify heat waves with consequences for human health, energy use and economic activity, including the tourist sector, which have yet received little attention.

**Fig. 1:**
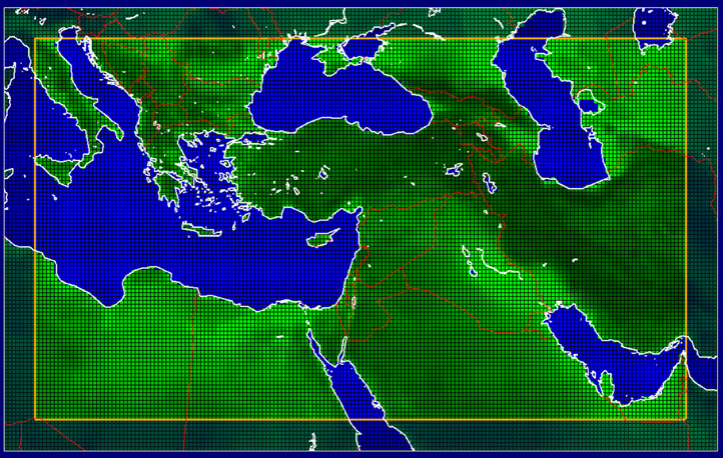
Geographic area and 0.22° latitude/longitude (~25 km) grid coordinates of the PRECIS regional climate model, applied for the period 1950–2099. The outer eight rows of grid cells are a “buffer zone” – not included in our analysis – to circumvent inconsistencies between the global and regional models

Here we present a comprehensive regional climate assessment as the EMME has only been marginally addressed by the Intergovernmental Panel on Climate Change (see Christensen et al. 2007). From a historical perspective this region is interesting as it is the cradle of civilization and has remained very significant since antiquity. The geographic area on which we focus is indicated in Fig. 1, depicting the domain of the climate model used in our study (section 2). We apply this EMME definition somewhat flexibly, dependent on the availability and quality of data, and also in view of national borders and model constraints. We discuss the data available for the assessment, and investigate the natural climate variability during the past 500 years based on natural proxies and documentary sources (section 3). Subsequently we analyze the climatology of the recent past for which instrumental data and re-analysis data sets are available (section 4). This includes trends and extremes of temperature and precipitation. We place the results into a global context by examining the role of large-scale circulation patterns and tele-connections in the Supplementary Online Material (SOM).

Sections 5 and 6 present projections of climate change for the 21st century based on the intermediate A1B scenario of the Special Report on Emissions Scenarios (SRES) of the Intergovernmental Panel on Climate Change (Nakićenović and Swart 2000). Our projections are based on the output of a regional climate model, which serves as a dynamical downscaling tool to refine the global climate simulations onto a finer mesh that more realistically describes the surface topography. In section 7 we summarize the potential impacts, supported by a literature-based analysis in the SOM, addressing fresh water resources, energy use, air quality, human health, terrestrial ecosystems including agriculture and fire risks, and the marine environment including sea level rise and ecosystems. Section 8 presents the conclusions.

## Data and model used

In much of the EMME meteorological observations are not publicly available. Therefore we use the Climatic Research Unit (CRU) TS3.0 data (http://badc.nerc.ac.uk/data/cru/). This dataset offers the most complete, consistent and updated compilation of gridded precipitation and temperature data at the global scale in general and for the Middle East in particular (Tanarhte et al. 2011). The data are stored at a spatial resolution of 0.5° latitude/longitude, and cover the period 1901-2006. The monthly time series of various climate variables include air temperature, precipitation, and vapor pressure, interpolated from surface observations. A more comprehensive description of this dataset can be found in Mitchell and Jones (2005).

We performed simulations using the PRECIS (Providing Regional Climates for Impact Studies) regional climate model, based on the United Kingdom (UK) Meteorological Office Hadley Centre HadRM3P model (Jones et al. 1995, 2004) for the 20th and 21st centuries. PRECIS applies the same formulation of the climate system as its parent Atmosphere-Ocean General Circulation Model (AOGCM), HadCM3 (Collins et al. 2005), which is also used to provide the lateral boundary conditions, and is driven by the IPCC SRES A1B emissions scenario. PRECIS accounts for the radiative forcings by changing concentrations of greenhouse gases, including ozone, and sulfate aerosols, and for interactions with the surface and deep soils.

According to the Fourth Assessment Report (AR4) of the IPCC (2007) the A1B scenario leads to a “best estimate” global mean temperature increase of 2.8°C with a likely range of 1.7 to 4.4°C, and a global mean sea level rise of 21–48 cm. In contrast, scenario A2 leads to a global temperature increase of 3.4°C with a likely range of 2.0 to 5.4°C, and a sea level rise of 23–51 cm. The A1B scenario can be considered as “intermediate” and assumes a future world of very rapid economic growth, global population that peaks in mid-century and declines thereafter, and the rapid introduction of new and more efficient technologies (Nakićenović and Swart 2000). The output of the HadCM3 model for the A1B scenario has been used and discussed in the IPCC 4th Assessment Report (Meehl et al. 2007, and Supplementary Material). The more pessimistic A2 scenario (higher greenhouse gas emissions) assumes that economic development is primarily regionally oriented and per capita economic growth and technological change more fragmented and slower than in A1B (IPCC 2007).

PRECIS was run for A1B over the period 1950–2099, applying a horizontal resolution of 0.22° latitude and longitude (about 25 × 25 km) and 19 vertical levels (Fig. 1). In section 4 we show that the simulation captures the mean climatic conditions and patterns, as well as the increasing temperature tendency observed in the 20th century. We present long-term temperature trends, comparing CRU (TS3.0) data with PRECIS output, including an overlap period between 1951 and 2006, and the projected 21st century changes (defined as differences relative to the control period 1961–1990) of temperature and precipitation for the 30-year intervals 2010–2039 (near-future), 2040–2069 (mid-century) and 2070–2099 (end-of-century). We mostly concentrate on the latter two periods.

## Climate variability during the past 500 years

**Fig. 2:**
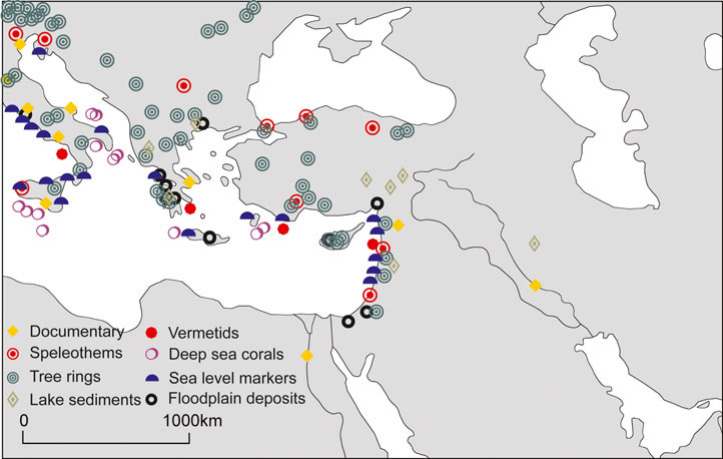
Locations of marine and terrestrial proxy data sources with seasonal to multi-decadal resolution. Note that the proxy lengths vary, provide different climate information and may record climate conditions during different seasons

Direct instrumental records of climate variables in the Mediterranean region are available for the past 1–2 centuries, and it is helpful to use indirect indicators, i.e.”proxy” data, to reconstruct earlier climate changes (Jansen et al. 2007; PAGES 2009). The information is derived from natural archives and documentary evidence (Brázdil et al. 2005). The Mediterranean is a region with a broad spectrum of proxy data, both in time and space that allow climate reconstructions over the past centuries. Figure 2 shows the locations for which marine and terrestrial proxies from the EMME are available.

The data sources are biased towards the northwestern part of the area and provide an indication of the potential of climate proxies to reconstruct climate variations at the local scale, and also have limitations in reconstructing the entire natural climate variability including inter-annual to multi-centennial variations. The lengths of the time series vary among the proxies. Proxies are also sensitive to multiple climate signals (e.g. temperature, precipitation, sea level changes, pH, sea surface temperature and water circulation). Furthermore, proxies are different in terms of temporal resolution (seasonal such as tree rings, to multi-decadal such as marine deep sea corals) and represent climate conditions during different parts of the year (Hegerl et al. 2011). Aspects such as sensitivity, reproducibility, local availability and continuity throughout time periods can differ as well (Mann 2002).

In the EMME the seasonality of rainfall is of major importance. There is a strong northwest-southeast gradient of the contribution of winter precipitation to the annual totals. In Italy winter precipitation typically accounts for 30–40% of the total precipitation. From southern Greece and Libya towards the Middle East the contribution of winter precipitation to the annual totals exceeds 50%. Over parts of Egypt, Jordan, Israel, Lebanon, southern Turkey and Cyprus, it reaches 60–80% (Xoplaki 2002). Here we present indications of past winter precipitation and summer temperature variations over the EMME during the past half millennium. We performed a multivariate calibration of the large-scale information in the proxy data network (documentary evidence, natural proxies) against 20th century gridded (0.5° resolution) land-based meteorological data (CRU TS3.0). Statistical relationships between the proxies and the instrumental data are applied to the pre-1901 period. Winter precipitation and summer temperature over the area 10°–40°E and 30°–45°N are reconstructed under the assumption of stationarity of the statistical relationships within the calibration period. Details about the methodology can be found in Luterbacher et al. (2004) for temperature and Pauling et al. (2006) for precipitation.

**Fig. 3:**
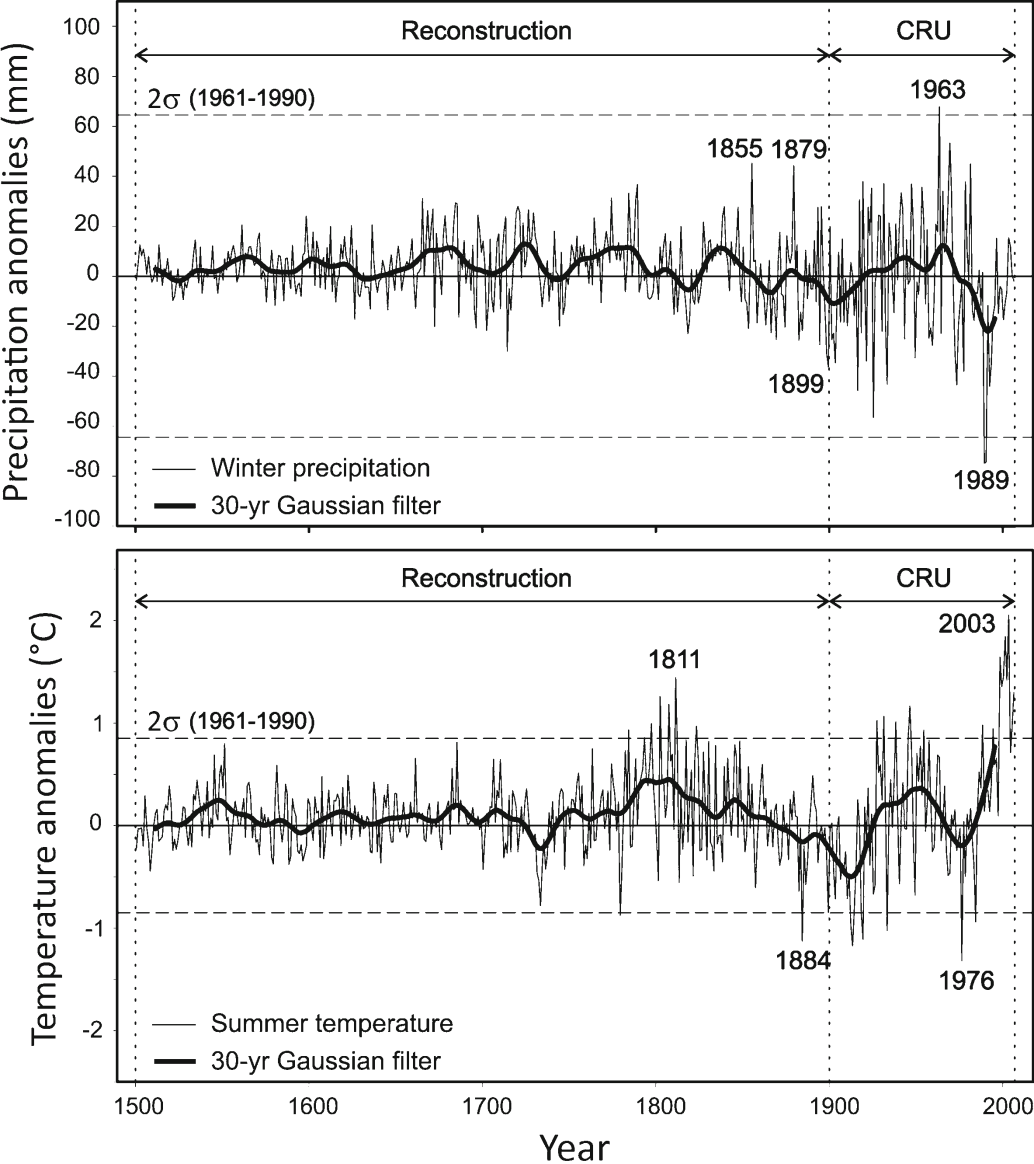
Winter (DJF) mean precipitation anomalies (top) and summer (JJA) mean temperature anomalies over land in the region (10°–40°E, 30°–45°N) from 1500 to 2006 (relative to 1961–1990). The data for the period 1500–1900 are from multi-proxy reconstructions, for 1901–2006 from CRU TS3.0. The thick black line is the 30-year Gaussian low pass filtered time series

None of the proxies shown in Fig. 2 directly represents December–February precipitation or June–August temperature conditions in the area. Therefore, the reconstructions are based on climate information and influences extrapolated from the Mediterranean and Europe. Figure 3 (top) shows the winter mean precipitation anomalies (relative to the reference period 1961–1990) from 1500 to 2006. The time series are composed of a reconstructed time period between 1500 and 1900 and the gridded CRU data for the period 1901–2006. The time series clearly indicate reduced variability prior to the early 19th century, partly due to the lower number of proxy data available. Consequently, the uncertainties can be substantial, especially for the first 2–3 centuries. Therefore, particular care is required when interpreting seasonal extreme values, especially prior to 1800, as the method tends to fit towards the 20th century mean conditions. Wetter and drier periods compared to the reference period 1961–1990 (186.5 mm average winter precipitation) can be identified in the pre-twentieth century period. There is compelling evidence of a strong drying trend starting in the early 1960s with lowest precipitation rates in the late 1990s. The likely driest (1988–89) and wettest (1962–63) winters, both, took place within the 20th century.

Figure 3 (bottom) presents the average summer EMME temperature anomalies over land of the period 1500–2006 (relative to the 1961–1990 mean of 29.7°C). The horizontal dashed lines indicate the 2σ standard deviations over the period 1961–1990. Summer temperatures from 1500 to around 1800 appear to have not been distinctly lower compared to the reference period. However, unlike for the winter precipitation, the full spectrum of variations cannot be captured. The warm phase around 1800 might actually be artificial due to possible inhomogeneities in the instrumental data before the mid-19th century and other factors, being subject of discussion (e.g. Frank et al. 2007). The 20th century is characterized by a strong increase in summer mean temperature until around the 1950s followed by a cooling trend until the mid-1970s. Subsequently, an exceptionally strong, unprecedented warming is observed, including the hottest summer on record in 2003, clearly exceeding the 2σ standard deviation of the period 1961–1990.

## Climate of the recent past

### Annual and seasonal temperature and precipitation: north–south contrasts

The 1961–1990 annual mean temperature (TM) patterns illustrate the different climatic zones within the EMME, from the cold and humid mountainous conditions, the typical Mediterranean climate, to the hot and dry deserts (Fig. S1). According to PRECIS, in agreement with the CRU data, the annual mean temperature ranges from about 0°C in mountainous areas to about 15°–17°C in coastal parts of the northern domain. The southern part of the region, i.e. North Africa and the Middle East, has notable different temperature conditions with a mean temperature range of 18° to 28°C. PRECIS successfully reproduces the annual mean patterns, sometimes providing more detail and sharper gradients owing to the higher resolution (~25 km) than the gridded CRU data (~50 km).

During winter (December through February) in the northern, mountainous areas of the EMME average temperatures below −5°C occur, while they typically reach 15°C in the south. The comparison between CRU and PRECIS indicates that the model is somewhat cold biased, up to several degrees (Fig. S1). In contrast, during summer (June through August) PRECIS is slightly warm biased compared to CRU. This is most pronounced in the Arabian Gulf region where PRECIS temperatures are several degrees higher than CRU. For the southern part of the region both datasets show temperatures from 25° up to more than 40°C. The discrepancies between PRECIS and CRU are generally largest in regions with pronounced topography, indicating that the difference in resolution between the two datasets plays a role, and in desert regions where measurements are scarce.

The contrast between the northern and southern EMME climate conditions is most evident from the maximum temperature (TX) patterns (Fig. S2). During winter, the maximum temperature in the north is about 10°C while in the south it reaches up to 25°C (not shown). Again, PRECIS appears to be slightly colder than indicated by CRU in coastal and mountainous regions, partly related to the difference in resolution. The warm summers in the EMME region are illustrated by the mean maximum temperature patterns for June–August in Fig. S2, showing that in the northern parts TX approximates 22°–30°C. Very hot summer conditions, with TX exceeding 40°C, appear in the Middle East and North Africa. Figure S2 confirms that PRECIS tends to exaggerate the high temperature conditions around the Arabian Gulf and North Africa.

Figure S2 also presents average minimum temperatures (TN) during winter. Once more, north–south gradients are strong. Sub-regions at elevated locations in the north have relatively low minimum winter temperatures of −10°C and annual TN close to zero, while it is typically 20°C higher in the south (not shown). The Italian and Greek Peninsulas have an annual average TN around 10°C, in the Middle East and North Africa it is about 15°C (not shown). The TN patterns by PRECIS adequately represent those of CRU although the model is slightly cooler especially in the Arabian desert (though CRU includes only few stations there). Winters can be quite severe in the western Balkans and in the mountainous areas of Turkey and Iran, with relatively low minimum temperatures. Winter TN can even fall below −15°C in continental parts of the region, whereas minimum temperatures above 0°C are typical of the milder coastal climate. During summer TN in the northern domain ranges between 10° and 18°C, while in the south it is 20°C and higher (not shown).

The sharpest climate gradients within the EMME are related to precipitation through the influence of major synoptic weather systems. The spatial distributions of seasonal precipitation (RR for rain rate) over the EMME are presented in Fig. S3 (DJF and JJA). Most precipitation occurs in winter and autumn, and the patterns during these two seasons are similar (not shown). The orographic triggering of rainfall appears to be important throughout the region, possibly resolved in greater detail by PRECIS than in the CRU data. During winter PRECIS is wetter than indicated by CRU. Because of the paucity of rain gauge data in many mountainous regions, however, it is not certain to what extent PRECIS overestimates precipitation. In both datasets, the dominance of local topography is evident from high winter precipitation amounts over the windward slopes (exposed to moist air masses) on the western Balkans (RR up to 500 mm during DJF only). Sub-regions at high elevation in Turkey also receive substantial precipitation, while further inland in the Balkans and Anatolia, and especially in the southern EMME rainfall is much less. For most of the region summer is the dry season, in contrast to continental Europe where thunderstorms and substantial precipitation rates are common. Only in some continental locations in the Balkans and the Caucasus convective storms in summer contribute significant amounts of rain, i.e. comparable to locations in Central Europe (Oliver 2005). In the southern part of the domain, rainfall deficits and summer dryness are commonplace.

The large north–south contrast is evident in both annual and seasonal rainfall patterns. In the northern EMME the average total annual precipitation during the 1961–1990 reference period ranges from approximately 500 mm in the east to more than 1,000 mm in the west. The Dinaric Alps in the western Balkans and locations along the southern coast of the Black Sea receive the most rain, averaging more than 1,000 mm/year. In other parts of the Balkan Peninsula, western Turkey and the western part of the Middle East rainfall amounts to about 600–700 mm/year. In Cyprus total annual precipitation is approximately 300–400 mm/year, while in North Africa and the Arabian Peninsula the total annual precipitation does not exceed 200 mm/year.

### Indices of climate extremes: hot and dry days, heavy precipitation

To assess extreme temperature and precipitation conditions in the EMME for the control period (1961–1990) we calculated several climate indices. Based on the characteristics of the EMME, emphasis is given to high temperatures, heavy rainfall and drought conditions. These indices, as defined by the World Climate Research Program (WCRP) (Peterson 2005; Klein Tank et al. 2009), were calculated using daily PRECIS output. Note that the CRU data cannot be used in this context as only monthly means are available. The indices are expressed as the annual occurrence of a parameter value exceeding a certain threshold: tropical nights, i.e. the number of days per year with minimum nighttime temperatures TN > 25°C; heavy precipitation days, defined as the number of days per year with precipitation RR > 10 mm, and the hot day index, i.e. the number of days with a maximum temperature TX > 35°C.

A slightly different set of indices was applied by Zhang et al. (2005), for example TX > 25°C for hot days and TN >20°C for tropical nights, which were selected because of their regular use for extra-tropical latitudes, but are less meaningful for regions with generally warm conditions. We prefer the higher values, also because they are more useful for comparison with our future projections (for which TX = 25° and TN = 20°C are common rather than extreme). The indices of Zhang et al. (2005) were derived from a dataset compiled during the regional workshop “Enhancing Middle East Climate Change Monitoring and Indices” (Sensoy et al. 2007), and unfortunately the original data are not publicly available. To nevertheless obtain indications of the performance of PRECIS, we calculated the same indices over the given number of years (within the period 1951–2003), presented in Table 1. In the last column we also show the number of consecutive dry days (CDD), defined as the maximum number of successive days with RR < 1 mm. Table 1 shows that we obtain rather good agreement, especially for the daytime maximum temperature, while the largest discrepancies occur for the heavy rainfall index.

**Table 1:** Comparison of indices of extremes between the PRECIS model and observations (Zhang et al. 2005). The indices refer to the number of days per year, averaged over the period for which observations and matching model data are available within the period 1951–2003. TX is the daytime temperature maximum, TN the nighttime temperature minimum, RR the rainfall rate and CDD the consecutive dry days

	TX > 25°C		TN > 20°C		RR > 10 mm		CDD	
	Obs	Model	Obs	Model	Obs	Model	Obs	Model
Heraklion	128	118	72	61	15	5	203	170
Nicosia	192	161	92	95	9	3	187	173
Mafraq	185	180	3	8	4	2	180	206
Riyadh	277	265	182	140	3	0	292	275
Dhahran	270	234	190	244	2	2	284	274
Palmyra	193	185	72	75	3	1	272	191
Istanbul	99	101	21	12	22	19	116	115
Ankara	104	80	2	7	11	17	114	181
Abadan	253	257	158	162	2	5	277	291

The geographical patterns of the selected indices, based on PRECIS output, are presented in Fig. S4. The top left panel presents the number of very hot days. Again the meridional gradient is very steep around 36°–38°N, and to its north the number of very hot days is less than a month/year. This geographical range coincides with the Fertile Crescent in the Middle East, between the Anatolian highlands and the Syrian desert. In contrast, in the southern part of the domain extended heat periods, i.e. 3–6 months/year with TX > 35°C are common. Further, tropical nights appear to occur during one month or less per year in the northern EMME, whereas in the south this is typically 1–2 months and up to more than 4 months per year around the Arabian Gulf. Note that in the Gulf region PRECIS may overestimate temperatures, and the latter number is probably an upper limit.

Subsequently we calculated the number of days with heavy precipitation. Along the western edge of the Balkan Peninsula and other high-elevation areas, e.g. in the Caucasus, heavy precipitation occurs about 40 days per year. About 25 heavy precipitation days per year occur over the Taurus mountain range in southern Turkey and the Zagros Mountains, which extend along southern and western Iran into northern Iraq. Finally the number of dry days (RR < 1 mm/day) has been derived, as presented in the bottom right panel of Fig. S4. The above mentioned wet regions have fewest dry days, i.e. 160 to 200 per year, while in lower-elevation and northern coastal Mediterranean regions this ranges between 250 and 300 per year. The driest areas are located in the southern EMME, with up to 300 dry days per year in several countries of the Middle East, e.g. Israel and Syria, up to a year in the desert areas of Egypt, Saudi Arabia and southern Iraq.

## Long-term temperature trends

The mean monthly temperature anomalies over the period 1901–2100, relative to the period 1961–1990, have been calculated based on both CRU data and PRECIS output. Whilst the next section focuses on the future projections, here we present the entire period, including the 1951–2006 overlap interval between RCM results and CRU data. We selected several major cities across the EMME domain, as we expect the observational basis of the CRU dataset to be most extensive and our analysis results most relevant for living conditions at these locations. The selection criteria furthermore include the geographical distribution, i.e. the representativeness in view of the various climatic conditions in the EMME. A map is presented in Fig. S5. The monthly mean temperatures during the period 1901–2006 were calculated from the CRU-TS3.0 dataset, while present and future data covering the period 1951–2100 were derived from the PRECIS results.

**Fig. 4:**
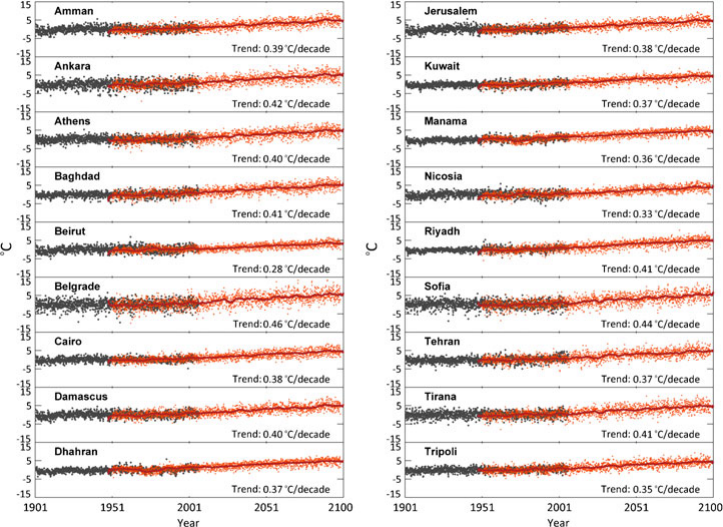
Time series of mean monthly temperature anomalies relative to the reference period (1961–1990) at 18 locations (Fig. S5). CRU data (*grey*) represent past and recent conditions; PRECIS data (*red*) represent recent and future conditions, and the red line shows the 5-year running average

The time series of monthly mean temperature anomalies at the 18 cities are shown in Fig. 4. The PRECIS results can be compared with the CRU data for the common period 1951–2006. It appears that PRECIS reproduces the CRU derived temperature anomalies well for most sites. PRECIS performs quite good both for sites with a relatively small seasonal temperature range, mostly located at low latitudes in arid regions (e.g. Cairo, Dhahran), and for stations at higher latitudes (e.g. Belgrade, Sofia) with a much more pronounced seasonal cycle. For Beirut, however, the magnitude of the seasonal cycle is underestimated and in Riyadh it is overestimated.

PRECIS appears to generally reproduce the temporal evolution of mean temperature anomalies in the EMME region. Linear regressions were calculated to infer the trends in mean monthly temperature anomalies from the PRECIS results, and the statistical significance was estimated using the Student’s *t*-test. The temperature changes are evident, and the rates of change per decade are indicated in Fig. 4. All trends are statistically significant at the 95% confidence level. Our findings are consistent with those of Zhang et al. (2005) and Alexander et al. (2006), who presented recent warming trends over the region. The temperature trends in the EMME typically vary from 0.28° to 0.46°C per decade. The largest increases appear in some continental locations such as Belgrade, Sofia, Ankara, Baghdad and Riyadh with trends in excess of 0.4°C/decade.

The same analysis was performed for maximum (TX) and minimum (TN) temperature anomalies (Table 2). For TX the largest upward trends are calculated for Belgrade, Sofia, Tirana and Ankara with 0.48°, 0.46°, 0.45° and 0.44°C per decade, respectively. The daytime maximum temperatures in Amman, Athens and Baghdad are found to increase by 0.40°C/decade. For TN, large positive trends exceeding 0.40°C/decade are derived for Belgrade, Riyadh, Baghdad, Athens, Sofia and Ankara. Interestingly, in the northern part of the EMME the maximum (daytime) temperatures TX increase relatively strongly, whereas in the southern part the minimum (nighttime) temperatures TN contribute most to climate warming. In general, most of these cities are already exposed to high temperatures in summer, and should anticipate exceedingly hot conditions. A more detailed analysis of our climate projections is presented in the next section.

**Table 2:** Linear trends (in °C/decade) over the period 1951–2100 from PRECIS output of the daytime mean temperature maximum (TX), mean (TM) and nighttime minimum (TN) in 18 cities

	TX	TM	TN
Amman	0.40	0.39	0.40
Ankara	0.44	0.42	0.41
Athens	0.40	0.40	0.42
Baghdad	0.40	0.41	0.43
Beirut	0.28	0.28	0.28
Belgrade	0.48	0.46	0.45
Cairo	0.36	0.38	0.40
Damascus	0.42	0.40	0.40
Dhahran	0.37	0.37	0.38
Jerusalem	0.38	0.38	0.39
Kuwait	0.35	0.37	0.39
Manama	0.36	0.36	0.36
Nicosia	0.34	0.33	0.34
Riyadh	0.38	0.41	0.44
Sofia	0.46	0.44	0.42
Tehran	0.37	0.37	0.39
Tirana	0.45	0.41	0.39
Tripoli	0.35	0.35	0.38

## Climate projections

### General conditions: strongest summer warming in the northern EMME

**Fig. 5:**
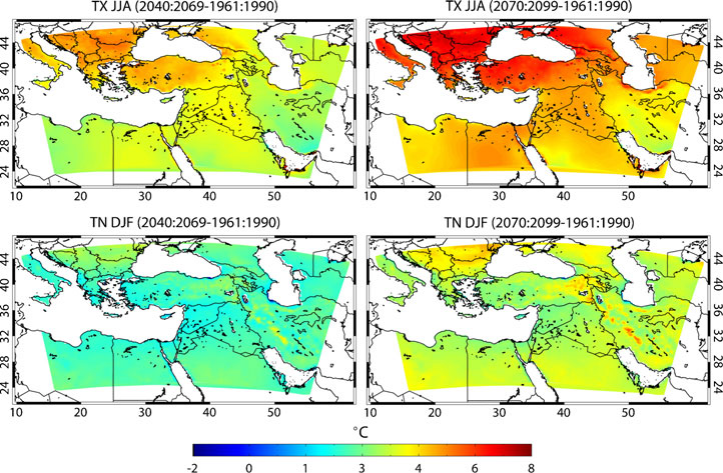
Patterns of changing mean summer maximum (JJA) and mean winter minimum (DJF) temperatures, TX (*top*) and TN (*bottom*), respectively, calculated from PRECIS output. The *left panels* show the mean changes for 2040–2069 and the *right panels* for 2070–2099 relative to the 1961–1990 control period

We first discuss the changes of daytime maximum (TX) and minimum (TN) temperatures in summer, the season with the largest – A1B scenario – projected warming in the region (Christensen et al. 2007; Hadjinicolaou et al. 2010; Giannakopoulos et al. 2010) and with potentially strong impacts. The results of the PRECIS projection, illustrated by Fig. 4 and for two periods in the top panels of Fig. 5, indicate that the regional warming will be gradual, both of TX and TN, ranging from 1°C to 3°C in the near-future (2010–2039), to 3–5°C in the mid-century period (2040–2069) and 3.5–7°C by the end of the century (2070–2099). In each period, this warming is more spatially uniform for winter TN, while for TX it is most pronounced at latitudes north of 36°–38°N (reaching 6–7°C in the Balkans, Turkey and the Caucasus by 2070–2099) and weaker in the southern EMME (~3.5°C in Libya, western Saudi Arabia and southern Iran). The relatively strong upward trend in the northern EMME indicates a continuation of the increasing intensity and duration of heat waves observed in this region since 1960 (Kuglitsch et al. 2010).

Further, in the southeastern EMME TN rises at least 1.5°C more strongly than TX by 2070–2099 as a consequence of increasingly cloudy conditions and reduced radiative cooling, notably in summer, which is most relevant for heat wave conditions (not shown). Figure 5 contrasts the projected increases in summertime TX (upper panels) and wintertime TN (lower panels). It highlights the exceptional trend in the daytime temperature maxima in summer, expected in the Balkans and Turkey, i.e. the region around the Black Sea. All results of TX and TN changes are statistically significant at the 95% confidence level by applying a bootstrap (Mudelsee and Alkio 2007).

The modeled changes in precipitation exhibit a large variability in space and time (Fig. S6). In winter the strongest drying is projected around the eastern Mediterranean, the coastal Levant and North Africa (−10% to −25% in 2010–2039, −20% to −35% in 2040–2069 and −30% to −50% in 2070–2099), consistent with the studies mentioned earlier. On the other hand, Italy, the Balkans, northern Turkey and the Caucasus may become wetter in winter during the 2nd half of the century, probably related to the increased evaporation from the surrounding water bodies. Most of these changes are not statistically significant at the 95% level, especially over the mountains of the western Balkans, southern Turkey, the Caucasus and Iran. In summer, the EMME drying is particularly large (greater during 2040–2069 compared to the end of the century) and interestingly, the areas that will experience winter increases are expected to dry in the spring and summer. Another notable feature is the projected, statistically significant, strong increase in precipitation throughout the southeastern part of the domain, related to the northward expansion of moist air masses from the tropics. Note, however, that these relative changes should not be over-interpreted since rainfall during the control period in the southern EMME is minor.

The projected changes in temperature are large and in agreement with previous work, and can be used with confidence in impact assessments. The eastern Mediterranean drying and the increasing precipitation patterns around the Gulf are also consistent with observed recent trends (Alpert et al. 2008), but must be interpreted with caution, owing to the large temporal variability of rainfall and the inherent limitations of climate models (global and regional) to simulate the hydrological cycle. Interestingly, in summer the meridional temperature gradient is waning, as the northern EMME warms more strongly than the south (e.g. Fig. 5).

### Indices of extremes: increasing heat stress

This section presents the indices of climate extremes as defined in section 4. For reference, the spatial pattern of the hot day index (TX > 35°C) of the 1961–1990 period is shown in Fig. S4. The calculated changes of the numbers of hot days per year are positive throughout the EMME and remarkably large and statistically significant in all periods (2010–2039, 2040–2069 and 2070–2099). By the end of the century the index increases by 0–15 days over the higher elevation areas and 20–40 days for most of the domain. The strongest increase (more than 2 months) appears over the Levant and the North African coast, approaching the conditions experienced in the Gulf region during the reference period. Interestingly, these changes do not parallel the summer TX shown in Fig. 5. The tropical night index (TN > 25°C) for the reference period is between 0 and 20 days per year for most of the region and 3–4 months around the Arabian Gulf (Fig. S4). A gradual future increase is projected (not shown), starting in the south of the EMME and progressing north, increasing by almost a month at the low-elevation areas of the Balkans, the Levant, the North African coast and southern Iran, and by 2 months and more in the southeastern part of the region (also including some locations around the Aegean Sea and Cyprus). The notable exception is the small increase over mountainous areas (less than 10 days).

It appears that the sub-regions for which the largest increases in the tropical night index (TN > 25°C) are expected may conversely experience the smallest increases of the hot day index (TX > 35°C); and the reverse also applies, as suggested above in the discussion of Table 2. Nevertheless, the overall heat stress can be very large. This applies especially to the Arabian Gulf region where hot and humid weather conditions already predominate in summer. Since tropical nights tend to exacerbate the adverse effects of daytime heat stress (Fischer and Schär 2010) the increase in the number of days with TN > 25°C by more than 2 months is of great concern.

**Fig. 6:**
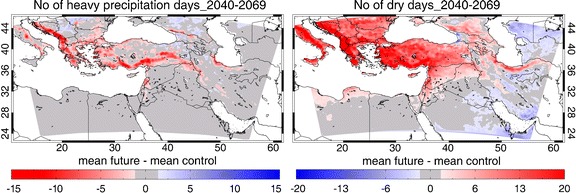
Patterns of changing number of days per year with heavy precipitation (RR > 10 mm, *left panel*) and number of dry days (RR < 1 mm, *right panel*), calculated from PRECIS output, showing the mean changes for 2040–2069 relative to the control period 1961–1990

Figure 6 presents the projected changes of precipitation related indices of extremes with a focus on the daily rainfall. The annual number of days with less than 1 mm precipitation for the 1961–1990 period exceeds 300 (>80% of the time) over the southern and eastern part of the domain (Fig. S4), demonstrating the near-absence of rainfall. In future this is not expected to change much apart from a small decrease in the number of dry days (up to ~10 days/year) around the Arabian Gulf.

In the northern EMME, mainly in the Balkans, Turkey, Cyprus, Lebanon and Israel, the number of rainy days may decrease, e.g. by 5–15 days at mid-century and by 10–20 days per year at the end-of-century. This appears to be a continuation of a trend observed in Greece since about 1960 (Nastos and Zerefos 2009). The modeled changes are associated with a reduction of cloudiness, which allows more solar radiation to be absorbed at the surface and contributes to the relatively strong increase of TX. The number of days with heavy precipitation (>10 mm/day) is expected to decrease in the high-elevation areas of the EMME (Fig. 6), the main locations of such events during recent climate conditions (Fig. S4). These changes are mostly statistically significant at the 95% level, except over some eastern parts of the domain (eastern Turkey, Caucasus, Iran). The intensity of precipitation (maximum amount of rain per day) is expected to decrease except over the northern Balkans and the Caucasus.

### Statistical distributions: hot summers in capital cities

**Fig. 7:**
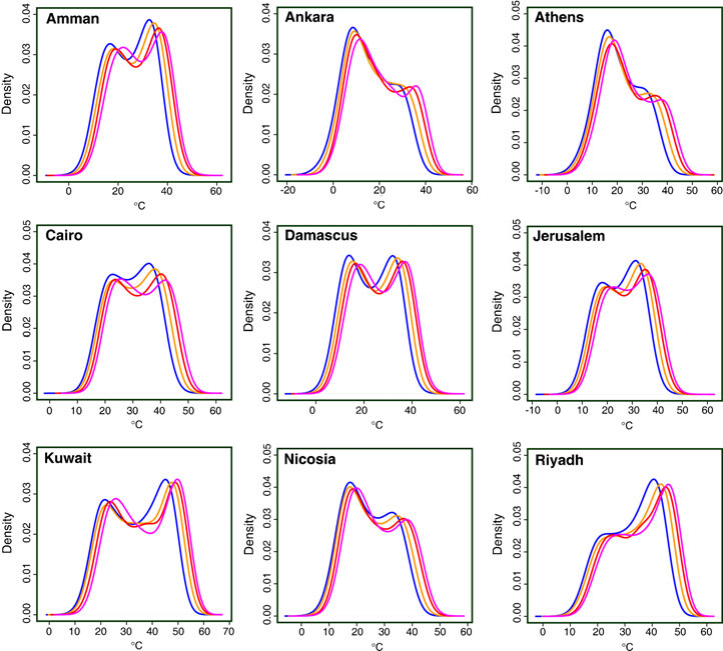
Probability density distributions of daytime maximum temperatures (TX) in the periods 1961–1990 (*blue*), 2010–2039 (*orange*), 2040–2069 (*red*) and 2070–2099 (*magenta*), calculated from PRECIS output

Figure 7 presents the kernel densities, i.e. the non-parametric estimates of the probability density functions (Wilks 2006), derived from the daytime maximum temperature (TX) calculated by PRECIS, for a selection of the capital cities within the EMME region. At all locations during the 1961–1990 period (blue line) the distributions have two peaks (bimodal), the first mode representing the cold (i.e. mild) season and the second the warm season (in agreement with observations; see Hadjinicolaou et al. 2010). In the northern and western EMME (e.g. Ankara, Athens, Nicosia) the first mode is higher, indicating the prevalence of cool and mild conditions during late autumn, winter and early spring. In the locations of capitals towards the eastern and southern EMME the second mode dominates, which illustrates the long duration of the warm season (Fig. 7). In the future periods, a gradual shift of the density curves to the right occurs, being most pronounced for the second mode. The peak heights decrease, though not very strongly. The changing tails of the distributions demonstrate the importance of increasing hot extremes, up to 5–6°C by the end-of-century.

Consequently, extremely high summer temperatures, which may have occurred only infrequently in the recent past (99% percentile of the 1961–1990 June–August distribution of modeled TX), for example 30°C in Beirut, 39°C in Athens, 47°C in Riyadh and 51°C in Kuwait City, are projected to become the norm by 2070–2099. We also calculated the occurrence of temperature anomalies (1°C interval) from the mean maximum temperatures (TX) in summer (June–August) during the control period 1961–1990 and the end-of-century (both relative to the control period) (not shown). In all locations, with the exception of Ankara and Tripoli (i.e. the capitals with largest temperature variability) the 2070–2099 and 1961–1990 distributions do not overlap, suggesting that the coolest summers at the end-of-century may be warmer than the hottest ones in the recent past. As an example, the hottest summer on record in Athens in 2007, during which the mean TX was 3.3°C higher than the 1961–1990 mean (Founda and Giannakopoulos 2009), would be among the 5% coolest ones by the end of the century.

## Potential climate change impacts

A critical aspect in the EMME will be the upsurge of extremely high summer temperatures. Hot conditions that occurred only rarely in the reference period may become the norm by the middle and the end of the century. The coolest summers during the period 2070–2099 may actually be warmer than the hottest ones during 1961–1990. Climate change in the region will have important negative consequences for humans and ecosystems, especially due to heat stress and reduced water resources; and population growth and economic development may aggravate the situation. In the subsequent paragraphs we discuss the climate impacts based on the preceding analysis as well as the literature review presented in the SOM.

Air quality is expected to become poorer in the EMME. Whereas human-induced emissions in most of Europe are decreasing, they are increasing in Turkey and the Middle East, which affect ozone and particulate air pollution, leading to excess morbidity and mortality. In the northern EMME increasing dryness will likely be associated with fire activity and consequent pollution emissions. Furthermore, the EMME has many large cities, including several megacities in which air quality is seriously degraded (e.g. Cairo, Tehran). There is compelling evidence that daytime maximum temperatures (TX) in the EMME are increasing, which is projected to continue in the future, leading to extended heat waves. While heat waves are defined relative to local conditions, it is evident that the level of discomfort is influenced by the already very high summer temperatures. Generally, heat waves are the type of weather extreme with most casualties. Excess mortality is greatest among sensitive groups such as people suffering from sickness, the elderly, children, and in general the urban population. Synergistic effects with related issues such as air pollution and water availability will need to be further investigated. This also applies to vector-borne parasitic and viral diseases, which have recently expanded into the EMME after many years of decline.

**Fig. 8:**
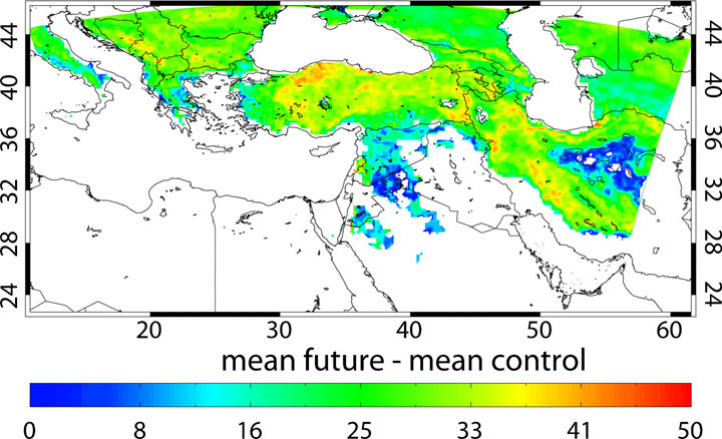
Changes in the length of the growing season in days/year for the period 2040–2069 relative to the control period 1961–1990, calculated from PRECIS output. Note that frost free areas appear *white*

The EMME has a high biodiversity, notably of plant species, related to the large gradients in topography, soil fertility and climate conditions. During the past millennia the conversion of natural ecosystems into croplands and overgrazing have already strongly shaped the land cover. The EMME encompasses sub-regions that are very suitable for agriculture, whereas others are not. Our PRECIS projections suggest that the milder winters in the north will be associated with a lengthening of the growing season by about one month per year by mid-century (Fig. 8). However, this advantageous influence will likely be overshadowed by the adverse consequences of extreme weather conditions; for example, the increasing number of very hot days (TX > 35°C) by 2–4 weeks/year, and the decrease of soil moisture.

The Mediterranean Sea can be seen as a small-scale ocean with a thermohaline circulation, intermediate and deep water transports. The eastern Mediterranean is an essentially land-locked basin with nutrient-poor surface waters (“marine desert”). In the past two decades rapid increases of the sea surface temperature have been observed, dominated by changes in summer. This corroborates our model results, indicating that temperature rise is more pronounced in summer than in winter. Modeling studies suggest that this tendency will continue in future, and the warming of surface and deep waters will result in salinization and water mass stabilization. The marine biodiversity can be affected, e.g. through reduced nutrient delivery to surface waters, “tropicalization” and the invasion of alien species through the Suez Canal.

Parts of the EMME, especially in the Middle East, are notorious for fresh water scarcity, and population growth and economic development are associated with a growing water demand (Chenoweth et al. 2011). We used the PRECIS projections to calculate changes in internal water resources. The model is less reliable in computing changes in precipitation than of temperature, though the results are robust, in agreement with previous work and consistent with our understanding of climate change mechanisms. Although a relative precipitation increase is projected for the southern part of the region, the absolute amount is insignificant, and negative effects dominate throughout the EMME. It is expected that the annual precipitation will typically decline by 5–25% in 2040–2069 and 5–30% in 2070–2099 relative to the reference period 1961–1990. The decreases will be particularly large (>15%) in Cyprus, Greece, Israel, Jordan, Lebanon, the Palestine territories and Syria. In combination with population projections of the UN for 2040–2069, per capita water resources may reduce by two thirds in Cyprus and Jordan, and by nearly half in Syria.

In the EMME fossil fuels dominate the energy supply, and their use is growing at a rate among the highest worldwide. Especially in the Arabian Gulf States the energy consumption for industry, traffic, air conditioning and desalination is burgeoning. This produces air pollution, including CO_2_, contributing to environmental and climate change. Our model results suggest that in the built environment during the warm season the need for space cooling will increase strongly. This includes intense cooling, expressed as the number of days/year at which air conditioning needs to achieve a temperature reduction of more than 5°C to reach a comfortable 25°C (i.e. cooling degree-days, CCD > 5°C) and alleviate the stress caused by heat waves. The projected mid-century increase of CCD > 5°C relative to the 1961–1990 control period is about 1–2 months (Fig. S7).

The energy use for air conditioning will grow in parallel with water deficits, which additionally distresses energy production by the need to desalinate sea water; and the competition with other sectors in need of water will increase. On the other hand, the heating requirements during winter will decrease in the northern part of the region, which moderates the overall increase in energy consumption and associated air pollution emissions. However, these compensating changes occur in different countries and seasons, and will not alleviate the need for extensive cooling during summer.

## Conclusions

Our analysis of meteorological data (CRU) for the 20th century and projections for the 21st century with the regional PRECIS climate model, based on the A1B scenario of the IPCC, indicate substantial climate changes in the EMME. From a 500-year historical perspective, derived from the combination of multi-proxy climate reconstructions and CRU data (past century), relatively dry and warm conditions are prevalent in the second half of the 20th century, with the strongest increase in summer temperatures since about 1990, consistent with the study of re-analysis data (Nastos et al. 2011). The evaluation of PRECIS output against the CRU data for the 20th century and the literature (Zhang et al. 2005) suggests that PRECIS realistically reproduces climatic patterns and indices of extremes.

The PRECIS results indicate a continual and gradual future warming, being strongest in the north. In comparison with the reference period (1961–1990) the mean temperature (TM) rise over land within the EMME will be about 1–3°C in the near-future (2010–2039), 3–5°C by mid-century (2040–2069) and 3.5–7°C by the end-of–century (2070–2099).

The mean temperature trend over the period 1950–2100, with a focus on capital cities in the EMME, is about 0.37 ± 0.9°C/decade. This suggests that the region warms much more strongly than other regions (IPCC 2007). We do not expect this to be an outlier result of PRECIS as the parent global model (HadCM3) has a temperature response to climate forcings close to the ensemble mean of 23 AOGCMs of the IPCC (Meehl et al. 2007), and it is also consistent with studies using other regional climate models (e.g. Önol and Semazzi 2009; Evans 2010).

The projected warming is approximately spatially uniform for nighttime minimum temperatures (TN), whereas the increase of maximum daytime temperatures (TX) is more rapid in the north, e.g. the Balkans and Turkey, than in the south. Towards the end of the 21st century, this will reduce the meridional temperature contrast by about 2°C.

The anticipated rapid warming in the EMME in the 21st century, especially during summer, combined with a general drying tendency, suggest important regional impacts of climate change. We recommend further investigations to study the effects on human health, the spreading of vector borne diseases, terrestrial and marine ecosystems, the availability of fresh water, agriculture and energy systems.

## Electronic supplementary material

Below is the link to the electronic supplementary material.

ESM 1 (DOCX 3343 kb)
