# Supplementary material for: Climate change and impacts in the Eastern Mediterranean and the Middle East
Source: Clim Change. 2012 Mar 7;114(3):667–87. doi: 10.1007/s10584-012-0418-4 (PMC4372776; doi:10.1007/s10584-012-0418-4)
Supplement: Supplementary file 1 — (DOCX 3343 kb) [file 10584_2012_418_MOESM1_ESM.docx]

**Climate change and impacts in the Eastern Mediterranean and the Middle East**

J. Lelieveld, P. Hadjinicolaou, E. Kostopoulou, J. Chenoweth, C. Giannakopoulos,

C. Hannides, M.A. Lange, M. El Maayar, M. Tanarhte, E. Tyrlis, E. Xoplaki

The Cyprus Institute, Nicosia, Cyprus

SUPPLEMENTARY INFORMATION

**S1 Atmospheric patterns and variability**

The proximity of the Eastern Mediterranean and Middle East (EMME) to the Atlantic and Indian Oceans and the extensive land masses of Eurasia and Africa, with maritime and continental air masses and diverse climatic characteristics, places the area at the crossroads of many global climate patterns. Most of the precipitation occurs during the boreal winter, when the mid-latitude zone of baroclinicity expands southwards. Then, the intra-seasonal and inter-annual climate variability is closely linked to processes of both tropical and extra-tropical origin, which ultimately influence the variability of the North Atlantic storm track. During the warm and dry summer period, the northward retreat of the weakened baroclinic zone leaves the region more “exposed” to the direct influence of tropical signals, primarily the dominant South Asian monsoon.

S1.1 Links to mid-latitude variability

The EMME is influenced by many important tele-connections that arise from intrinsic modes of mid-latitude variability. The North Atlantic Oscillation (NAO) is a prominent mode that represents the North Atlantic jet and storm track variability (Hurrell et al. 2003). It consists of a large scale rearrangement of atmospheric air masses between the northern and subtropical Atlantic regions, and is sometimes seen as the regional manifestation of the hemispheric Arctic Oscillation (Thompson and Wallace 1998).

During the positive NAO phase (NAO+) anomalous northerly flow around the intrusion of the strong Azores anticyclone towards the central Mediterranean induces colder conditions, and the opposite during the NAO‒ (Xoplaki 2002; Trigo et al. 2004). Negative relationships between the winter temperature and winter NAO index are found in instrumental temperature data in Egypt (Hasanean 2004) and in coral proxy data for areas near the Red Sea (Felis and Rimbu 2010). The NAO impact on winter precipitation in the EMME is rather complex. During NAO+, the synoptic-scale activity is diverted towards northern Europe. In the Mediterranean a southwest-northeast division emerges with drier conditions in the west and north, including the northern Balkans and large parts of Turkey (Trigo et al. 2004). As a result the NAO index is negatively correlated with station precipitation in Greece and Turkey mainly during winter and partly in the autumn (Feidas et al. 2007). This is associated with reduced river discharge to the Mediterranean basin in Turkey (Struglia et al. 2004) and the flow of the Tigris and Euphrates. On the other hand, the positive NAO tendency in the 1990s was accompanied by a flow decrease of these rivers (Cullen and de Menocal 2000).

On the other hand, in the extreme southeastern parts of the basin the surface pressure is lower during NAO+. The presence of an upper level trough extending from the north triggers cyclogenesis over the warm waters of the region, e.g. Cyprus lows, being enhanced by the northerly advection of moist Mediterranean air (Feliks et al. 2010). This increased precipitation is restricted to the southeastern Mediterranean (Xoplaki et al. 2004; Dunkeloh and Jacobeit 2003), accompanied by increased river discharge in this area (Struglia et al. 2004), but does not reach the hyper-arid regions of central Libya, Egypt and the northern Red Sea (Felis and Rimbu 2010). Also, the frequency of cyclones during NAO+ is found to increase in the southeastern basin (Nissen et al. 2010). Cyclonic activity and precipitation events reduce towards the western and northern Mediterranean, while they become more frequent during NAO‒ (Raible 2007; Pinto et al. 2009). In parts of Algeria, southern Italy, Greece and some northern parts of the Middle East the correlation of precipitation and the NAO is not significant (Xoplaki 2002).

The climate variability in the EMME is also controlled by other circulation modes than the NAO (Qian et al. 2000). The Eastern Atlantic/Western Russia (EAWR) pattern consists of a wave train of anomalies extending from the North Atlantic to central Asia, with two primary centers located over southwestern Russia and western Europe. During its positive (negative) phase an anomalous northerly (southerly) flow dominates over the EMME, bringing colder (warmer) and wetter (drier) conditions in a wide zone from Malta towards Syria and further to the north over eastern Turkey (Krichak and Alpert 2005; Trigo et al. 2006). The regional dipole pattern of the hemispheric EAWR mode over Europe is also referred to as the North Sea Caspian Pattern (Kutiel et al. 2002).

The Eastern Atlantic (EA) pattern resembles a “southward shifted” version of the NAO pattern, reflecting modulations in the subtropical ridge intensity and location, however its impact on the EMME is marginal (Trigo et al. 2006). The Scandinavian pattern corresponds to the Eurasia-1 pattern and in its positive (negative) phase is associated with a dominant positive (negative) geopotential height anomaly over Scandinavia and two geopotential height anomalies of opposite sign over western Europe and central Asia (Xoplaki 2002). During its positive phase, the Scandinavian anticyclone bears striking similarity to blocking (Tyrlis and Hoskins 2008a). During winter in the Scandinavian sector it features a maximum across the Northern Hemisphere with almost 25% of the winter days associated with blocking (Tyrlis and Hoskins 2008b). During these conditions the mid-latitude westerly flow is obstructed and the storm track splits into a branch towards the Arctic and one towards the Mediterranean, bringing relatively wet conditions to the western and central Mediterranean and the western boundary of the EMME (Xoplaki et al. 2004). The influence of blocking on the temperature in the EMME is only weak.

S1.2 Links to tropical dynamics

The El Niño/Southern Oscillation (ENSO) is a prominent coupled ocean-atmosphere mode of inter-annual climate variability, originating from sea surface temperature (SST) anomalies in the tropical Pacific Ocean (warm El Niño/cold La Niña) and an associated atmospheric signature consisting of a pressure oscillation (Southern Oscillation) across the Pacific basin (e.g. Wang et al. 2004). The proposed mechanisms responsible for transferring the signal from the equatorial Pacific to mid-latitudes include standing modes across the tropical band that subsequently affect higher latitudes (Alpert et al. 2006). During warm ENSO events the Atlantic Hadley circulation and associated trade winds are weak and this can introduce a signal in the NAO through the Azores anticyclone (Wang et al. 2004; Brönnimann 2007). An alternative route involves the influence of ENSO through changes in the structure of the Pacific Hadley Cell circulation and the location of the Pacific/North American pattern (Huang et al. 1998). Subsequently, mid-latitude Pacific pressure deviations may excite wave trains that can induce anomalies downstream over the North Atlantic and Europe by affecting the position of the mid-latitude jet and the storm track (Alpert et al. 2006).

Although the ENSO effects across the Pacific Ocean and the surrounding regions are generally recognized, the impact on the EMME region is not fully understood related to the varying methodologies used, the intra-event variability and the non-stationary behavior of the source signal across the Pacific. Also, the signal can be masked due to non-linear interactions with extra-tropical variability. During winter there is no significant ENSO signal in the Mediterranean storm track intensity, however near the Aegean Sea the storms can turn towards the Black Sea, bringing wetter (drier) conditions over northwestern (southern) parts of Turkey (Kadioğlu et al. 1999). Although a large part of the eastern Mediterranean experiences dry winter conditions during ENSO warm events, rainfall may increase in Israel and the Jordan River discharge during October-March, attributed to the southern position of the subtropical jet, guiding depressions to the area (Alpert et al. 2006). It should be noted though that the above period includes the autumn season when, unlike winter, and during El Nino (La Nina) events, wet (dry) conditions dominate over the southeastern Mediterranean and the Middle East (Brönnimann 2007). This zone of abundant autumn precipitation under El Niño covers the Iberian Peninsula, northern Africa and the Middle East as far as Iran (Alpert et al. 2006). During spring EMME precipitation appears to be influenced by the northward (southward) displacement of the Mediterranean storm track during El Niño (La Niña) bringing dry (wet) conditions, mainly in the western part of the region, e.g. southern Greece (Munoz-Diaz and Rodrigo 2005). Apart from the remote and indirect ENSO effects, a tropical signal can directly influence the EMME climate through the synoptic-scale Red Sea troughs, tropical intrusions and dust storms from the Sahara desert (Alpert et al. 2006).

The EMME is also directly influenced by the massive South Asian monsoon and all processes that influence its variability. In summer, a dominant circulation in the EMME is the persistent Etesian northerly flow that serves as a ventilating system and a thermostat of the area (Ziv et al. 2004). The combined influence of the higher pressures over the western Mediterranean and the Persian trough extend from the Asian Monsoon trough towards the Mediterranean. Along with the surface flow, the monsoon induces large scale subsidence over the central and eastern Mediterranean, inhibiting convection and resulting in clear skies. This subsidence is sometimes attributed to the descending branch of the Hadley circulation over Africa and is linked to the large subtropical desert zones in the subtropics (see Rodwell and Hoskins 1996 for a discussion). Alternatively, it is seen as a part of a “Walker” type circulation with the ascending motion over the southeastern Asian (Ziv et al. 2004). Such a closed circulation was not identified by Rodwell and Hoskins (1996). The latter showed that the Asian monsoon heating induces a Rossby wave pattern to its west that leads to descent and forms a warm structure that expands to the west. The interaction of this warm structure with mid-latitude activity produces the large scale descent over the Mediterranean, whereas the orography is responsible for locking and intensifying the pattern over specific areas (Rodwell and Hoskins 1996). The vertical and horizontal structure of the summer circulation over the EMME and the role of the monsoon are further examined by Tyrlis et al. (manuscript in preparation).

S1.3 Regional climate projections

Although the PRECIS model projections do not provide information about future changes of large-scale tele-connections affecting the EMME, some aspects can be attributed to prominent patterns such as the NAO. For example, the winter drying across parts of Italy, the Balkans and Turkey – and especially upwind where orographic effects can be ruled out – is typical of an intensification of NAO+ when the storm track shifts to higher latitudes. At the same time, the prevalence of southerly flow in the northern part of the domain during NAO+ can explain the relatively rapid winter warming compared to the southern EMME. Further, a possible intensification of ENSO, especially El Niño events, may contribute to the northward displacement of the storm track. During summer, both the Etesian northerly flow at the surface, which acts as a ventilation system for the area, and the large scale subsidence, which inhibits convection and leads to adiabatic warming of the tropospheric column, are largely controlled by dynamical interactions between the South Asian monsoon and mid-latitude weather systems. The question whether the projected summer changes in the temperature and precipitation over the EMME region are related to alternations of the Etesian flow or the large scale subsidence in a changing monsoon environment requires further research.

**S2. Air quality**

S2.1 Recent pollution emissions

Atmospheric pollutants are hazardous to human health and ecosystems, both by episodic peak levels and the long-term exposure to relatively moderate enhancements. The American Heart Association states that each 10 µg/m^3^ increase of fine aerosol particles with a diameter of less than 10 µm (PM10) increases the relative risk for daily cardiovascular mortality by about 0.4-1% (Brook et al. 2010). Many gaseous and particulate pollutants are photochemically formed within the atmosphere from emissions by traffic, energy generation, industry, the burning of wastes and forest fires. In summer the EMME is largely cloud-free, and the relatively intense solar radiation promotes the photochemical formation of ozone (O_3_). It is produced by the oxidation of reactive carbon compounds such as carbon monoxide (CO) and volatile organic compounds (VOC), catalyzed by nitrogen oxides (NO_x_=NO+NO_2_).

In Europe, pollutant emissions have decreased in the past decades, indicated both by emission inventories and satellite measurements, whereas they have increased in the Middle East (Stavrakou et al. 2008; van der A et al. 2008; de Meij et al. 2011). The emissions have decreased relatively less in the Mediterranean member states of the European Union (EU). In Portugal, Spain and Greece the anthropogenic emissions of VOC and NO_x_ have actually increased and those of sulfur dioxide (SO_2_) have decreased only moderately. In fact, five Mediterranean EU countries contribute nearly half to the EU25 air pollution emissions (Lelieveld 2009). Furthermore, the growing emissions by international shipping add significantly to air quality deterioration in coastal areas (Marmer and Langmann 2005).

S2.2 Aerosol haze

Satellite measurements underscore the extended scale of high aerosol burdens, both of natural and anthropogenic origin (Papadimas et al. 2009). The fine aerosol particles of less than 1µm (PM1) are mostly composed of sulfates and particulate organic matter (POM), whereas the coarse particles (PM10) are dominated by desert dust, as well as sea salt in coastal regions and islands (Kouvarakis et al. 2002; Lelieveld et al. 2002). Measurements in Greece indicate rather high pollutant concentrations by long-distance transport of air pollution; high levels of SO_2_ have been attributed to coal burning in Central and East Europe (Mihalopoulos et al. 1997; Kallos et al. 1998; Formenti et al. 2001). Over desert regions synoptic disturbances and localized convection can mobilize mineral dust particles that often reach the EMME.

The pollutant sources include fire emissions. Around the Black Sea, in Bulgaria, Romania, the Ukraine and Russia, air pollution from biomass burning can be very strong, especially in summer, and reach the eastern Mediterranean within a few days (Sciare et al. 2003). Uncontrolled fires around the Mediterranean – often human-ignited – contribute strongly to the aerosol burden, especially during dry spells. Black carbon, which can have strong climate effects, originates from biomass and fossil fuel combustion in similar amounts (Sciare et al. 2003). The relatively large mass fraction of POM includes many oxygenated hydrocarbons, indicative of aged and photochemically processed air pollution and biomass burning (Hildebrandt et al. 2010).

The combination of natural and anthropogenic particles forms an extensive haze over the EMME. Calculations with a global chemistry-climate model (Pringle et al. 2010) indicate annual mean PM10 concentrations of about 100 µg/m^3^ in the Middle East and even higher levels in North Africa, strongly influenced by mineral dust, whereas in southern Europe and Turkey they are typically 20-30 µg/m^3^ (Fig. S8). Generally the concentrations are lowest in winter (due to rainout). During summer, e.g. in Cyprus and Crete, remote from pollution emissions, the local aerosol concentrations are close to the EU air quality standard for particulate matter (PM10) of 55 µg/m^3^. It appears that the fine aerosol mass (PM1) is about 80-90% anthropogenic, whereas about 60-80% of the coarse particle (PM10) mass is natural. Recent aerosol trends over the Mediterranean are negative whereas they are positive over Turkey and the Middle East (Papadimas et al. 2009; de Meij et al. 2011). Scenario calculations for the mid-century period suggest that PM1 levels in this region will increase substantially due to increasing emissions of SO_2_ and NO_x_ (Fig. S8). The emissions may increase especially strongly in Turkey, which is expected to enhance air pollution throughout the Middle East.

S2.3 Ozone smog

In the Mediterranean region ozone often exceeds the EU eight-hourly air quality limit of 110 µg/m^3^, particularly in summer. Annually, the EU plant protection threshold (80 µg/m^3^) is exceeded more than 80% of the time. The relatively high O_3_ levels in background air indicate that Mediterranean ozone is among the highest in the world. The pollution can be lofted to 1-4 km altitude by land-sea breeze circulations, shallow convection and orographic flows (Millán et al. 2000). Above the boundary layer a stable reservoir layer channels air pollution south- and eastward. Over North Africa and the Middle East this layer is broken up by convection and turbulence, which mixes the pollution to the surface.

It is difficult to control the ozone smog, e.g. in the populated areas along the coast, because the local emissions add to an already high background. The highest ozone levels occur over the Arabian Gulf. Fig. S9 shows global O_3_ during summer, and the inserts the regional concentrations during July and August (Lelieveld et al. 2009). The wind arrows indicate the importance of pollution transport from Europe and the Mediterranean, channeled over the Gulf in the Persian trough, and strong local emissions from traffic, energy generation and industry are superimposed. Satellite measurements confirm the very high levels of ozone precursors (e.g. NO_2_) in this region, as well as a strong upward trend (van der A et al. 2008; Lelieveld et al. 2009). Based on the scenario simulations for the 21^st^ century it seems likely that ozone will continue to increase and that the EMME is a persisting air pollution hot spot.

S2.4 Megacity air pollution

According to the CIA World Factbook 2010 ([www.cia.gov/library/publications/the-world-factbook](http://www.cia.gov/library/publications/the-world-factbook)) population growth rates in southern Europe population are largely negative (zero to –2%/yr), whereas in the Middle East and North Africa they are positive (1-2%/yr). Population growth is associated with urbanization; and megacities (>10 million inhabitants) have become a common phenomenon, also in the EMME. In developing countries, urbanization is typically accompanied by air quality deterioration. Urban air pollution is estimated to cause about 800,000 premature deaths per year worldwide, mostly due to cardiovascular and respiratory diseases, and traffic emissions play a dominant role (Uherek et al. 2010).

Emission inventories suggest that ozone precursor sources in Tehran, Cairo and Istanbul are still among the middle-to-low range among the 30 largest cities of the world, although an upward tendency is expected (Butler et al. 2008). Gurjar et al. (2010) estimated strong health risks by particulate matter (PM10), applying the air quality guidelines of the WHO (2006). This analysis suggests that Cairo ranks among the megacities with poorest air quality in the world, leading to an excess mortality of about 14,000/year. Since population growth and urbanization in the southern and eastern parts of the EMME will likely continue, the situation will aggravate until appropriate measures are implemented. It has been shown that vehicle exhaust controls can successfully reduce the emissions of ozone precursors and particulate matter. It is anticipated that in the long-run traffic emissions may stabilize and possibly decrease by the middle of the century (Uherek et al. 2010).

S2.5 Effects of climate change on air quality

Although atmospheric chemistry-climate feedbacks can be important, e.g. by aerosols, the effects of future climate change on pollutant concentrations are rather uncertain. For example, it is difficult to predict changes in the removal rate by precipitation, which is important for soluble gases and aerosols (Zeng and Pyle 2003; Raes et al. 2010). Overall, emission controls (or the lack thereof) have a stronger influence on air quality than climate and land-use changes (Brasseur et al. 2006; Ganzeveld et al. 2010). The growing fire risk will lead to increasing pollution emissions by forest fires, which will also depend on abatement policies and their enforcement (Giannakopoulos et al. 2009a). Airborne mineral dust may either increase or decrease, depending on changes in vegetation cover and on meteorological conditions (Millán et al. 2005; Tegen et al. 2004). Different models give different results, and especially the effects of anthropogenic land-use change are debated. A study in Israel indicates an increasing trend of several dust-days per decade during the period 1958-2006, related to atmospheric transports from Africa, though the relation to climate change is unclear (Ganor et al. 2010). In future the expected warming and drying may enhance energy use for air conditioning and desalination and associated pollution emissions. Furthermore, the atmospheric conditions will probably become more conducive for ozone formation, especially during heat spells (Confalonieri et al. 2007; Guerova and Jones 2007).

**Human health concerns**

Climate change affects human health directly and indirectly through multiple scales and complexities, and on different time horizons (Paz 2006). The impacts vary geographically in relation to environmental and climatic conditions and the vulnerability of the local human population. There is growing evidence that the EMME already experiences impacts of climate change on public health (Confalonieri et al. 2007). For example, in the past decades the frequency and intensity of heat waves and the duration of drought periods in the Balkans and Turkey have increased significantly (Kuglitsch et al. 2010).

Confalonieri et al. (2007) anticipate that climate change will affect the health status of millions of people, particularly those with low adaptive capacity, through:

• Increases in malnutrition and consequent disorders, with implications for child growth and development;

• Increased deaths, disease and injury due to heat waves, floods, storms, fires and droughts;

• The increased burden of diarrhoeal diseases;

• The increased frequency of cardio-respiratory diseases due to higher concentrations of ozone and particulates related to changing pollution emissions and climate change;

• The altered spreading of infectious disease vectors.

S3.1 Heat waves

Heat waves can have profound impacts on human health (Haines et al. 2006; Knowlton et al. 2009; Anderson and Bell 2011), on power systems and regional economies, ecosystems (Ciais et al. 2005) and agriculture (Battisti and Naylor 2009). Studies of the human vulnerability to extreme heat and in general of the temperature-mortality relationship have identified a variety of factors such as age and health conditions, socioeconomic status, housing conditions, prevalence of air conditioning and social aspects (e.g. clothing), which can vary strongly with climatic zones and the level of socioeconomic development (McMichael et al. 2006).

During heat waves, excess mortality is greatest among the elderly and people suffering illnesses, who are confined to bed, living alone and those being heavily exposed, i.e., directly below the roofs of buildings (Vandentorren et al. 2006). Much of this mortality is due to cardiovascular, cerebrovascular and respiratory diseases. Further, it is likely that children will suffer disproportionately from climate change (Ebi and Paulson 2007). Urban populations are relatively strongly affected because of the heat island effect and the reduced ventilation in the built environment, leading to higher temperatures than in the surroundings. Heat waves may also increase air pollution concentrations as they typically occur during stagnant conditions with strong ozone formation, which may further enhance the death rates.

Heat waves can be defined as spells of at least six consecutive days with maximum temperatures exceeding the local 90^th^ percentile of the reference period 1961-1990 (Fischer and Schär 2010; Zhang et al. 2005). The ten most severe ones in the EMME since 1950 were reported in the period 1987-2006 (EM-DAT, The International Disaster Database, http://www.emdat.be; WHOSIS Global Health Observatory; [http://www.who.int/gho](http://www.who.int/whosis/en/index.html)). A case study in Greece indicated that the excess mortality almost doubled during a major heat wave in 1987, and more strongly in Athens than in smaller cities and non-urban areas, while the number of hospital admissions in the greater Athens area increased almost fivefold (Katsouyianni et al. 1988; 1993).

Comprehensive studies of heat wave related health impacts have been performed for the year 2003, when about 15,000 excess deaths occurred in France alone, and 25,000-70,000 in Europe, including Portugal, Spain, Italy, Switzerland, Croatia and other countries (D’Ippoliti et al. 2010; Haines et al 2006; Robine et al 2006). In France the summer of 2003 was the hottest on record with a mean temperature of 3.6°C above the long-term climatology (Battisti and Naylor 2009). Some of the health impacts may have been related to air pollution as the heat wave was accompanied with exceptionally high ozone concentrations at the surface, related to suppressed ventilation of the boundary layer (Guerova and Jones 2007).

Our climate projections for the 21^st^ century suggest that heat waves will become more common in the EMME. Figs. 5 and 7 illustrate that TX will rise most strongly during summer, and may even increase by ~6°C in 2070-2099. Further, the increased occurrence of tropical nights (TN>25°C) by more than two months per year in the southern EMME (not shown) will exacerbate the heat stress, particularly for vulnerable groups (e.g. infants, elderly) who live in urban areas with high levels of air pollution, as the prolonged heat can exceed the physiologic adaptive capacity of humans.

S3.2 Vector-borne infectious diseases

During the long history of humanity in the EMME, the region has repeatedly been distressed by epidemic infections, in particular vector-borne parasitic and viral diseases such as Malaria, Dengue Fever, Dengue Hemorrhagic Fever, Leishmaniasis and others. After several decades of steady and major decline, mainly due to integrated vector control policies, a recent upsurge of these diseases occurred. Moreover, vector-borne diseases such as West Nile Fever, Chikungunya Fever and Crimean-Congo Hemorrhagic Fever, leading to epidemics in Africa, have spread northward and have become an emerging threat to the EMME (G Christophides, personal communication). For these diseases, the distribution and abundance of vector organisms and intermediate hosts are important, affected by various physical and biological factors. Integrated studies indicate that increasing temperatures may lead to a worldwide advance of vector organisms and the spreading of infectious diseases (McMichael 2003). Temperature related changes in the life-cycle dynamics of the vector species and pathogenic organisms promote the potential transmission of these diseases (Paz and Albersheim 2008).

S3.2.1 Malaria

This illness is transmitted to humans through the bites of *Plasmodium* parasite infected female *Anopheles* mosquitoes (WHO 2010). Although the spreading of Malaria involves multiple factors, it is considered to be a highly climate-sensitive tropical disease (Patz et al. 2008). Reiter (2001) concluded that the epidemic risk in the Mediterranean is likely to be small and easily controlled. However, recent studies rather indicate that the EMME offers a favorable climate for sporadic cases of autochthonous Malaria (Doudier et al. 2007). Many parts of the eastern Mediterranean were previously endemic Malaria areas, and the disease was eradicated only fifty years ago (Schwartz 2005). Several countries in the EMME are again facing the Malaria problem, including Turkey (Alten et al. 2000) and Israel (Kopel et al. 2010), related to climatic and socioeconomic factors, regional proximity to areas of resurgent Malaria transmission and health systems (Wieliczko and Staroniewicz 2010).

The spreading of Malaria cannot be related to a single cause and it will be necessary to study the links between seasonal temperature, rainfall and the vector distribution, as well as anthropogenic influences (e.g. water management, land-use and mosquito control activities) and entomological risk factors (Ponçon et al. 2007).

S3.2.2 West Nile Fever

The West Nile virus is also vector-borne and causes a zoonotic illness of the type of the Japanese encephalitis serogroup of flaviviruses. The virus is primarily transferred by mosquitoes that attack several types of vertebrates, including birds and mammals (Paz 2008). It is often transmitted between birds by the mosquitoes of the *Culex* genus, which tend to breed in foul standing water in drains and catch basins (Epstein 2001).

In many parts of the EMME West Nile Fever is endemic (Reiter 2010) and infections in vertebrates may have occurred for centuries (Marr 2003). Paz (2006) and Paz and Albersheim (2008) demonstrate a correlation between the West Nile virus occurrence and climatic factors. It appears that in Israel most cases of West Nile Fever occur in metropolitan areas and in a narrow area close to the seashore, where the heat stress is enhanced by high humidity. The cooler climate in the north of Israel possibly prevents the further spread of the virus, which could possibly change in future.

Most studies of the spread of West Nile Fever in the Mediterranean find that extreme weather contributes importantly (e.g. Bernabeu-Wittel 2007; Feki 2005; Reisen et al. 2010). However, there are also other factors such as poverty, population dynamics, the inappropriate medical and agricultural use of antibiotics, and local environmental change, which promote mosquito breeding and could spawn outbreaks in areas once the virus has established itself (Epstein 2001).

S3.2.3 Dengue Fever

The presence of the Dengue vector mosquitoes *Aedes aegypti* and *Aedes albopictus* has been confirmed in many areas of the EMME in the past two decades (Fakeeh and Zaki 2003; Pacsa et al. 2002; Rathor 2000). Rathor (2000) considers the EMME as an area where Dengue Fever is re-emerging after 50 years of absence, although the observational data base to support this is thin. The re-emergence of Dengue Fever in connection to climatic conditions is controversially debated. Although a warm climate is a necessary condition for the vectors (Burnett and Matthews 1997), the relationships between meteorological factors, the vector ecology and Dengue transmission have yet to be determined (Hartley et al. 2002). Although there are a number of statistical models correlating climate change with the frequency of Dengue epidemics, predictive regional models are still in their infancy (Pongsumpun et al. 2008).

S3.2.4 Leishmaniasis

This is a major vector-borne illness and the only tropical disease that has been endemic to southern Europe for decades (Dujardin et al. 2008). Several cases have been reported in the EMME since the 1990s and epidemiological studies indicate that the disease is re-emerging (Jaffe et al. 2004). Studies of the effects of climate change on Leishmaniasis have concentrated on parameters that influence its vector, the sand fly. Its spreading is largely controlled by temperature and humidity (Lindgren et al. 2004). Further, Ready (2010) proposes a direct relationship between climate change and the Leishmaniasis distribution through the influence of temperature on the parasite development in female sandflies, as well as indirect effects of environmental changes on the abundances of the vector species. The emergence or re-emergence of Leishmaniasis in the EMME may contribute to its spread into Europe (Ready 2010).

S3.2.5 Chikungunya Fever

Chikungunya Fever (CHIK) is a viral disease transmitted by the *Aedes* mosquitoes. The first transmission of the virus in Europe occurred in the summer 2007 in northeastern Italy and has been linked to the high density of *Aedes albopictus* (Tiger mosquito). Favorable conditions include mild winters (e.g. January temperature ≥ 0°C), an average annual rainfall of about 500 mm/year and summer temperatures of 25-30° C (Straetmans et al. 2008). The European Centre for Disease Prevention and Control indicates that the risk of CHIK in Europe is to be taken seriously (Tilston et al. 2009). However, the transmission of CHIK has also been associated with visitors from hotspot areas (e.g. North Africa).

S3.2.6 Crimean-Congo Hemorrhagic Fever

The Crimean-Congo Hemorrhagic Fever (CCHF) is one of the most widely distributed tick-borne diseases worldwide, also affecting people in the EMME (EPISOUTH 2008). It has been suggested that the optimum conditions for ticks and their life cycle are increasingly fulfilled in the region through influences of climate change on the vegetation structure (Gale et al. 2008). Mild weather conditions in winter and spring promote the tick reproduction and can influence its spreading. For example, a CCHF outbreak in Turkey has been linked to the relatively mild spring in the previous year (Semenza and Menne 2009; Vorou et al. 2007).

Estrada-Peña and Venzal (2007) performed a model study and found that rising temperature and decreasing rainfall in the Mediterranean region can lead to a sharp rise of the suitable habitat areas for the *Hyalomma* ticks (i.e. the principal vectors of CCHF) and their spread to the north, with the highest impact at the margins of the current geographic distribution area. Climate change is not solely responsible but rather a co-factor with several other aspects such as land-use and ecological changes, agricultural policies, route changes of migratory birds, socio-economic changes and others.

**S4 Terrestrial ecosystems and agriculture**

The EMME is characterized by strong orographic variations, especially in the north and east, whereas the south is much less topographically pronounced. Combined with soil fertility and climate gradients, this gives rise to both low-water and high-temperature limiting conditions for vegetation growth across much of the region. The natural vegetation is dominated by mixed forests (broadleaf and needle-leaf) in the west, grasslands and shrubs in the south and east, and dry barren areas further south (Huston 1994; Olson and Dinerstein 2002). Further, human action has strongly shaped the EMME land cover through the conversion of about half of the vegetated areas to croplands (ECJRC 2004; Hoekstra et al. 2005) and the degradation of grass- and shrub-lands by overgrazing (Le Houérou 2002).

S4.1 Agriculture

Climate conditions in the EMME allow for a large variety of crops, including C3 and C4 cereals, legumes and root crops (Leff et al. 2004). Fruit trees such as citrus, olive, almond and grape are also widely grown, especially along the Mediterranean coast (Moriondo et al. 2008). Crop production in some countries, such as Egypt, Libya and most of the Arabian Peninsula, is almost entirely dependent on irrigation. The EMME encompasses many sub-regions where climate and soil conditions vary from being very suitable for crop production, e.g. in the northwest, to poorly suitable in the south and east. Developing countries are often very vulnerable to climate change because of their dependence on agriculture and the structural difficulties to adapt (Nelson et al. 2009; Hertel et al. 2010). Since many countries in the EMME have limited capital resources, while facing rapid population growth, the capacity of agriculture to ensure the future regional food security is critically challenged.

Climate change can affect agriculture directly through the meteorological conditions that influence crop growth and yield. We used the PRECIS output to calculate the growing season length, defined as the number of days between the last spring frost and the first autumn frost. The model results indicate that by mid-century the length of the growing season may increase by about one month per year in Turkey, the Balkans and part of Iran (Fig. 8). Some crops, such as winter wheat, will profit from the milder winters whereas others, such as sunflower, are more prone to heat stress (Moriondo et al. 2010). Most importantly for crop cultivation, climate change will likely be associated with a higher frequency of extreme weather conditions. Our model results suggest that in these sub-regions the occurrence of very hot days (TX>35°C) will increase by 2-4 weeks per year by mid-century, which could even seriously damage high-temperature tolerant crops grown in the region (e.g. corn and sorghum). Heavy precipitation events will increase by several days per year in the northern part of our domain (e.g. Balkans, N-Turkey; Fig. 6), whereas their number decreases in southern Turkey and Greece. Further, the increase of evapotranspiration and the decrease of soil moisture in dry areas can substantially reduce the land suitability for some major crops in the EMME (Hegazy et al. 2008). These results corroborate the findings of Kitoh et al. (2008) and Evans (2010), indicating that the future rainfall decrease in winter will strongly affect the eastern Mediterranean coastal areas and endanger the Fertile Crescent in the Middle East.

In summer, rain deficits can enhance the intensity of heat waves because the barren soils tend to lose their ability to cool by evaporation and thus emit more sensible heat (Vautard et al. 2007). Actually, a cascade of processes can intensify both the reduction of soil moisture and the heat stress, thus aggravating agricultural losses. In a warmer climate soil moisture decreases more rapidly in spring by enhanced evaporation and reduced snowmelt, which was shown to occur in southeastern Europe (Rowell and Jones 2006). This can linger into the summer and reduce rainfall, which accelerates the drying (Schär et al. 1999; Koster et al. 2003). Observational evidence in southeastern Europe has confirmed the viability of this mechanism, suggesting an amplification of temperature extremes due to soil moisture deficits (Hirschi et al. 2011).

Nevertheless, the effects of climate change on agriculture are associated with important uncertainties. Using output from two different climate models (CSIRO and NCAR), it appears that in subtropical regions either crop yield decreases or increases are possible. According to the NCAR model output the yield of rainfed maize and soybean could decrease by about 46% and 76%, respectively, by mid-century (Nelson et al. 2009). Conversely, based on the CSIRO model output the crop yields may increase by 62% for maize and 26% for soybean. El Maayar et al. (manuscript in preparation) investigated the yields of C3 and C4 cereal crops in the Mediterranean region since 1960, and could not establish a significant correlation with climate parameters, in spite of statistically significant changes in temperature and precipitation.

Overall, the future effects of climate change on crop production are expected to be negative, at least for the major crops in the EMME. This could have important consequences for countries with economies that are dependent on agricultural production and where the adaptative capacity is limited. But the uncertainties hamper policy making, independent of the capacity of agricultural systems to adapt to climate change. These uncertainties are not only due to climate change projections, but also to limitations of crop models. For example, links between the carbon and nitrogen cycles are not yet adequately accounted for (El Maayar and Sonnentag 2009). According to Lobell and Burke (2008) the influence of temperature changes is poorly characterized, although negative correlations between crop yields, heat waves and droughts are manifest (Battisti and Naylor 2009). Further, Ainsworth et al. (2008) and El Maayar and Sonnentag (2009) show that models have difficulties reproducing the fertilization effect of elevated ambient CO_2_ on C3 crop yields. While it is generally accepted that C3 crops benefit from elevated atmospheric CO_2_, it is unclear how climate change and increasing atmospheric CO_2_ will influence invasions of pests, insects and weeds (Chen and McCarl 2001; Rosenzweig et al. 2002; Long et al. 2009; Zavala et al. 2008).

S4.2 Natural ecosystems

The Mediterranean part of the EMME is one of the world regions with highest plant diversity (Huston 1994; Cowling et al. 1996; Dallman et al. 1998). It is, however, also an area where the threat to biodiversity by extensive habitat conversion and limited environmental protection has reached critical proportions (Hoekstra et al. 2005). Klausmeyer and Shaw (2009) reported that by the end of the century the typical Mediterranean climate may extend into the Anatolian and Balkan peninsulas, and could expand in the north and shrink in the south along the Tigris river basin. By considering synergetic interactions between climate change and other drivers Sala et al. (2000) predicted that among the major biomes on Earth, the Mediterranean biodiversity could decrease most strongly. By considering assemblages of plants, birds and mammals, it is expected that climate change will lead to reductions of phylogenetic diversity in southern Europe, whereas gains may be expected in northern Europe (Thuiller et al. 2011).

The increased frequency, intensity and duration of extreme climate events could have dramatic effects on the natural vegetation and may critically disrupt the balance between pathogens and vegetation, which can influence the life cycle of trees and whether or not they reach their full size (de Dios et al. 2007). A case study in Europe during the 2003 heat wave suggested a 30% reduction of gross primary productivity (Ciais et al 2005). In the EMME the vegetation currently at the rim of its natural distribution, e.g. in the alpine transitions in northern Italy and along the semi-arid/arid ecotone in the eastern Mediterranean, is expected to be most sensitive to climate change (e.g. Garcia-Romero et al. 2010).

Field studies have provided insight into the effects of droughts on Mediterranean forests. Extended droughts in the 1990s have severely weakened trees, increased their susceptibility to pathogens, and caused the death of populations (Montoya 1995). It appears that deciduous trees were generally more seriously affected than conifers. Another observational study showed that under drought conditions the natural *Quercus ilex* (Holm Oak; an evergreen broadleaf species) might have reduced access to soil phosphorus, adding to the overall stress (Sardans et al. 2008). Based on results of a long-term study (16 years) Henkin et al. (2010) reported that in the eastern Mediterranean dry years tend to favor thistles and crucifers while wet years tend to favor legume grasses. Interestingly, grassland showed a high resilience to climate variations through the buffering effect of its perennial herbaceous component against dramatic changes in community structure, which ultimately reduces the ecosystem vulnerability to climate change. Increased dry spells will likely promote fire events in dry regions (Moriondo et al. 2006; Good et al. 2008), as observed in Greece (Dimitrakopoulos et al. 2011), which may contribute to a reduction in soil organic matter and vegetation productivity (Diaz-Delgado et al. 2002). It is unclear if the vegetation in the EMME can adapt to the increased fire frequency, even though fire has long been considered a dominant ecological and evolutionary factor (Riera et al. 2007).

S4.3 Links with air pollution

Several studies of the effects of ozone on plants indicate that the projected increase in near-surface O_3_ will likely exacerbate the negative effect of climate change on crop production, biodiversity and forest growth (Volk et al. 2005; Wittig et al. 2007; Long et al. 2009). Holland et al. (2006) estimated that for the year 2000 the global economic loss due to arable crop damage by ozone was equivalent to 2% of the agricultural production. Interestingly, Mediterranean countries (and Germany) suffered the biggest part of this loss, related to the high levels of ozone. Sitch et al. (2007) argued that the reduction in carbon uptake by terrestrial vegetation following an increase of atmospheric ozone could constitute a positive feedback on global warming. Collins et al. (2010) found that on a 20-year time scale the impact of ozone damage to vegetation significantly enhances climate change by the reduced CO_2_ uptake, whereas on longer timescales (e.g. 50 years) the effect declines due to recovery of the vegetation. Several studies have confirmed the negative effects of high ozone on tree growth and photosynthesis, and on the competitive ability of plant species (The Royal Society 2008). Since ozone, heat stress and droughts are expected to concurrently increase in the EMME, it will be important to investigate the synergistic effects.

**S5 Marine environment**

The Eastern Mediterranean (EM) is a relatively large basin (1.65×10^6^ km²) that includes the Ionian and Aegean Seas and the Levantine Basin. It is essentially land-locked, with water exchange occurring primarily through the narrow and shallow Strait of Sicily (~1 Sverdrup, Sv = 0.001 km^3^/s; Béranger et al. 2005). Water movement across the Sicily Strait is anti-estuarine, with Atlantic-derived water flowing eastward at the surface, and more dense intermediate waters flowing westward over the sill at depth (~360 m). Much lower water exchange also occurs at the Dardanelles Strait (Tzali et al. 2010) and Suez Canal (Abril and Abdel-Aal 2000).

Confinement of surface circulation within the EM drives complex basin scale, sub-basin scale, and mesoscale dynamical features (Hamad et al. 2006; Malanotte-Rizzoli et al. 1997; Özsoy et al. 1993; Robinson et al. 1991). The basin scale circulation is largely cyclonic, with Atlantic water mixing with Levantine Surface Water (SW) moving east- and northward around the Levantine shelf and flowing through the eastern Cretan Arc into the Aegean. The Levantine SW then mixes with Black Sea water and flows cyclonically around the Aegean coast. Superimposed on this large-scale pattern is a dense network of meanders and permanent or semi-permanent mesoscale features, e.g. anticyclonic eddies and gyres (Pelops eddy, north Ionian Sea; Ierapetra eddy, southeast of Crete; Shikmona gyre region and Cyprus eddy, southeast of Cyprus) and cyclonic gyres (Rhodes gyre, north Levantine). Although the knowledge of EM surface circulation progressed rapidly after the 1980s POEM program (Physical Oceanography of the Eastern Mediterranean), the complexity of the EM dynamics and lack of sampling in southern regions indicates the need for still more observational and modeling efforts.

S5.1 Thermohaline circulation

Surface waters of the EM are subject to intense heating, and, as evaporative losses are greater than precipitation and river inputs, concentration processes drive the thermohaline circulation of the basin. A major component of this circulation is Levantine Intermediate Water (IW), which forms in the Rhodes gyre area during winter when salty water is cooled by dry northerly winds and vertical stability is reduced (Lascaratos and Nittis 1998; Malanotte-Rizzoli et al. 2003). The Levantine IW subsequently spreads throughout the EM at depths of 200-400 m and is a major contributor to outflow waters at the Strait of Sicily and in the western Mediterranean. The EM thermohaline circulation is also affected by Cretan IW, formed in the Aegean Sea north of Crete, and eastern Mediterranean Deep Water (DW) formed in the southern Adriatic Sea.

The surface and thermohaline circulation of the EM contributes to the ultra-oligotrophic nature of this marine environment. Deep water pools within the EM are low in nutrients (nitrate ~6 µM; Krom et al. 2005) vs. comparable ocean regions (e.g. nitrate ~40 µM; Karl et al. 2001), in part because EM source waters are derived from nutrient-poor surface Atlantic waters and are subject to biological activity during its transit through the western Mediterranean. Thus, despite large winter mixed layer depths in the EM (> 90 m), nutrient concentrations within this layer of biological activity remain low and limit new production (Siokou-Frangou et al. 2010). Such low nutrient availability and plankton biomass has caused the EM to be described as a “marine desert”, with productivity highest in frontal regions (e.g. Zervoudaki et al. 2007) and at deep convection sites (e.g. Siokou-Frangou et al. 1999).

S5.2 Recent changes

The Mediterranean Sea is relatively small and its waters have a high turnover rate (~100 years), thus it is sensitive to environmental change (Béthoux et al. 1999) and has shown clear signs of response to climate forcing in past decades (Lionello et al. 2006). Warming and salting of western Mediterranean DW has been observed by many authors (e.g. Rohling and Bryden 1992; Tsimplis and Baker 2000). This change has probably resulted from the climate-driven alteration of the evaporation – precipitation (E–P) balance in the Mediterranean, damming and the reduction of river inputs, and recent changes in Mediterranean DW formation rates and composition (Millot et al. 2006; Skliris et al. 2007). Recently, warming and salting of the western Mediterranean DW over the past century (0.0047°C per decade and 0.006 psu per decade) have been linked to warming of surface air temperatures in the northern hemisphere (Vargas-Yáñez et al. 2010a). If only recent decades are considered (1943-2000), the rate of western Mediterranean DW warming is higher (0.02°C per decade).

In the eastern basin, increasing temperature and salinity of DW (0.04°C per decade and 0.01 psu per decade) were calculated by Manca et al. (2004). The observed abrupt change in the EM thermohaline circulation during the 1990s provides a dramatic example of climate impacts on the marine environment in this region. Roether et al. (1996) showed that warm and dense salty waters from the Aegean had displaced DW of Adriatic origin at the bottom of the eastern basin. This new source of dense bottom water (Cretan DW) began forming in the Cretan Sea in 1987, and production peaked in 1991-1992 with a total volume of more than 3 Sv delivered by 2001 (Roether et al. 2007). Several explanations of this abrupt change, called the Eastern Mediterranean Transient (EMT), were proposed, including salinization due to reduced precipitation, reduced Black Sea outflow or river runoff, cooling due to severe winters in the early 1990s, and changes in wind stress (Roether et al. 2007). Most recently, application of an 1/8° eddy permitting ocean model with high-resolution atmospheric forcing indicated that the triggering elements of the EMT were atmospheric cooling (heat loss) and increased wind stress during winter in the early 1990s (Beuvier et al. 2010).

The EMT is also an example of how climate-driven change in circulation characteristics can affect marine ecosystems. The intrusion of Cretan DW into the Ionian and Levantine basins led to an increase in bottom water O_2_ content (because these DWs were newly formed) and uplifted the older water masses such that waters at intermediate depths in the basin (> 800 m) became more nutrient-rich (Kress et al. 2003). Several studies have explored the biological response to these physical and chemical changes. Oxygen utilization rates in bottom and intermediate waters of the eastern basin increased twofold following the EMT, likely due to greater availability of labile dissolved organic carbon at these depths (Klein et al. 2003). Mesozooplankton biomass at depth increased by up to two orders of magnitude after the EMT, and species found only in the northernmost margins of the Mediterranean before this event (i.e., *Calanus helgolandicus*) suddenly became abundant in the Levantine deep waters.

Over the past century, significant warming trends of the Mediterranean surface waters have been detected (CIESM 2008; Vargas-Yáñez et al. 2010b). In the eastern basin, increasing sea surface temperatures (SSTs) have been observed over the past two decades in the Aegean Sea and the Levantine (CIESM 2008; Romanou et al. 2010). The increase in eastern SSTs has been rapid (0.5-0.6°C per decade) and is dominated by temperature change during the summer (Nykjaer 2009), which is consistent with our model prediction that seasonal temperatures increase relatively strongly during summer. Nevertheless, it is not yet clear to what extent the SST increase in the EM is dominated by surface forcing or has been affected by recent changes in the thermohaline circulation.

S5.3 Future climate change

Climate warming of the western and eastern basins will continue to dominate the evolution of the Mediterranean through the 21^st^ century. Coupled Atmosphere-Ocean Regional Climate Models (AORCMs) predict an increase of 2.5-3°C in Mediterranean SSTs by the end of the century. For example, the modeling study of Somot et al. (2008), based on the A2 scenario, indicates an overall SST warming of 2.6°C (0.27°C per decade), with the maximum change (2.9°C) in summer and autumn. In the eastern basin, temperatures may increase by 2°C (Levantine Basin, winter) to 4°C (Northeast Aegean Sea, summer). Warming trends predicted by another AORCM, under scenario A1B (Artale et al. 2010), are of the same magnitude (0.16°C per decade), and both are considerably larger than warming trends observed over the past century in the Mediterranean Sea (Artale et al. 2010; Vargas-Yáñez et al. 2010a).

Warming of Mediterranean surface and deep waters will result in salinization, water mass stabilization and sea level change. Marcos and Tsimplis (2008) compared the results of twelve AOGCMs for the Mediterranean, and found a general warming and salting of the Mediterranean of 1.2 psu by the end of the century. For the eastern basin, Somot et al. (2008) predict an increase in salinity of 0.3-0.5 psu in the Levantine, and 0.8-1.0 psu in the northern Aegean Sea, with concurrent stabilization of water masses. This stabilization will result in a decrease in deep water formation, particularly in the Gulf of Lions and in the Levantine Basin (-20% deep and intermediate water formation; Somot et al. 2006). Newly formed deep and intermediate waters will moreover be less dense, with a maximum decrease in density in the Levantine Basin (‒0.98 kg m^-3^).

Finally, changes – and uncertainties – in predicted salinities will also have a strong effect on Mediterranean Sea level. Basin-wide increases in sea level are predicted, but will be dominated by strong spatial variability and significant increases due to the thermal component in the last 50 years of the 21^st^ century (Artale et al. 2010). The AORCM study based on the A2 scenario by Tsimplis et al. (2008) suggests a sea level rise of 13-25 cm in the 21^st^ century with lower rates in the EM than in the western Mediterranean. This is less than the global mean of 23-51 cm estimated for the A2 scenario (IPCC 2007), related to the compensating effects of thermal expansion and salinization, the latter being most significant in the EM.

Climate-driven change in water temperature, thermohaline circulation and sea level are certain to affect ecosystem function in the Mediterranean over the next century, in particular impacting ecosystems in the few EM “oases” where circulation dynamics and wind-induced upwelling support relatively high productivity and fish yield (Bakun and Agostini 2001; Siokou-Frangou et al. 2010). Higher water temperatures, changes in EM thermohaline circulation, reduction in nutrient delivery to surface waters, and overall “tropicalization” of these locations in the EM will be the first-order drivers of ecosystem change. These impacts will be compounded by secondary effects such as the movement of alien species from outside marine systems as EM circulation changes and temperatures rise. Near-shore water temperatures are predicted to increase by 2 to 4°C in the Levantine Basin by the end of the century (Coll et al. 2010), encouraging the movement of “Lessepsian” migrant marine organisms through the Suez Canal from the Red Sea. Winter 15°C isotherms have shifted northward along the Strait of Sicily in recent years, thus supporting the invasion of warm-water species from the eastern to western basins, which will continue in future. Marine biodiversity in the EM will be particularly susceptible to climate-driven change in the 21^st^ century, with impacts predicted to increase even within the next 10 years (Coll et al. 2010).

**S6 Water resources**

There is little doubt that climate change is associated with a drying of subtropical regions, including the Mediterranean and the Middle East (Gibelin and Déqué 2003; Held and Soden 2006; Mariotti et al. 2008; Gao and Giorgi 2008; Evans 2009). Part of the EMME is already notorious for water scarcity, and the demands on limited water resources by population growth and economic development are increasing. Here we investigate the changes in water resources based on PRECIS output. Water resources management typically occurs on a national and multi-national basis, hence an analysis of the effects of climate change must consider countries which are wholly encompassed by the model domain and which do not receive significant cross-boundary water inflows from trans-national rivers originating outside the modeled area. Therefore, we consider the impacts of climate change on the water resources of 15 countries (Table S1). For details about our methodology and additional results we refer to Chenoweth et al. (2011).

S6.1 Impact on precipitation and water resources

Internal renewable water resources (IWR) refer to those generated entirely within the boundaries of a country, thus being determined by the land area, precipitation and evapotranspiration rates. The total water resources of a country, however, also include cross-boundary flows. We used the ArcMap Geographical Information System and the PRECIS model calculated precipitation changes for 2040-2069 (mid-century) and 2070-2099 (end-of-century) relative to the 1961-1990 (recent) period. For example, using PRECIS precipitation output for the recent period we estimate an annual precipitation for the Gaza Strip of 122 mm per year. Being a very small territory of 360 km^2^, compared to the 625 km^2^ resolution of the PRECIS grid, and not fitting wholly within a single cell of the model output, the Gaza Strip spans three cells. Roughly 30% of the territory occurs within a grid cell with 126 mm precipitation, 30% occurs within a cell with 177 mm precipitation, and 40% with 82 mm. The weighted average for the territory is 122 mm of precipitation per year. The small size of the Gaza Strip and other small countries like Bahrain are problematic when calculating annual precipitation and IWR as the territory relative to individual model cells can significantly influence the results. Nevertheless, for our assessment the relative changes in precipitation are most relevant.

The calculated changes in precipitation in each country are used to estimate changes in IWR, which are assumed to change by the same percentage as the precipitation. This assumption is likely to produce a somewhat optimistic estimate of IWR since higher temperatures could be expected to result in a decrease of the proportion of the rainfall that converts into internal water resources. In semi-arid catchments the streamflow elasticity is typically high, and a 1% change in annual precipitation typically leads to a 2.0-3.5% change in annual streamflow (Chiew 2006).

Our model results suggest that the mean annual precipitation in the EMME will decline by 10-20% in 2040-2069 relative to 1961-1990. Using the FAO (2009a) AQUASTAT database as the baseline, mean precipitation thus declines from 454 mm in the control period to 396 mm in 2040-2069, although this decline is not uniform across the region and three countries are projected to receive increased precipitation. However, while the precipitation increase projected for Bahrain, Kuwait and Qatar exceeds 30%, the absolute amount is insignificant. Nine countries are projected to have a mean precipitation decrease of more than 10%, while both Cyprus and Lebanon will have a decrease in precipitation in excess of 20%. Based on the assumption that IWR change in line with precipitation across the region as a whole, they will decrease from 388 km^3^ to 343 km^3^. Table S1 shows the modeled effects of climate change on water resources for the study countries for 2040-2069 and 2070-2099.

The PRECIS results for the end of the century suggest a small additional decline in annual precipitation across the EMME, i.e. 2% relative to mid-century and 15% relative to 1961-1990. Three countries (Bahrain, Kuwait and Qatar) are projected to have a precipitation increase, but again the absolute size is insignificant. Eleven countries will experience a decrease in precipitation of more than 10% relative to 1961-1990 and four countries decreases of more than 20% (Greece, Jordan, Lebanon and the Palestinian territories). Across the region as a whole, IWR will decline from 388 km^3^ in 1961-1990 to 336 km^3^ in 2070-2099. Such significant precipitation and water resource decreases will require important economic and social adjustment across the region.

S6.2 Impact on international river basins

While there are several major trans-boundary river basins in the EMME region, due to data limitations only two can be evaluated here: the Tigris-Euphrates and the Jordan River basins. The Euphrates River is 3,000 kilometers long with a basin area of 879,790 km^2^. Based on the estimates of the FAO (2009a) the volumetric contributions of each of the riparian nations of the Tigris-Euphrates have been calculated, which suggest that the annual discharge averages 73.4 km^3^ (Chenoweth et al. 2011). The PRECIS output indicates that the average annual Tigris-Euphrates River discharge could decline by almost 10% by 2040-2069 relative to 1961-1990. The decline is greatest in Turkey at 12%, while only 4% in Iraq. A further decrease in river discharge is expected by 2070-2099, however, this is less than 1%.

The Jordan River is 250 kilometers long with a catchment area of 18,500 km^2^ (FAO 2009b). The catchment upstream of Lake Tiberius contributes approximately 0.58 km^3^ of water to the basin (Murakami, 1995). Downstream of Lake Tiberius, the Yarkmouk River contributes 0.4 km^3^, with the remainder of the east bank of the Jordan River contributing about 0.2 km^3^; another 20% (0.11 km^3^) of the lower Jordan discharge comes from Israel, with the remainder of the river discharge (0.21 km^3^) coming from the West Bank (Murakami, 1995). The PRECIS simulations indicate that available water resources in the Jordan River basin will decline during the course of this century, suggesting a 22% reduction by 2040-2069 and 30% by 2070-2099 (compared to 1961-1990), with the decrease being relatively even across the catchment.

These end-of-century decreases in overall river basin discharge are less than those predicted by Kitoh et al. (2008) who estimated a decrease of 29-73% for the Euphrates and 82-98% for the Jordan River for 2080-2099, depending upon the climate change scenario considered. However, the projections of Kitoh et al. (2008) were questioned by Ben-Zvi and Givati (2008) who pointed at large discrepancies between simulated and observed values for the Jordan River system. The PRECIS modeled decline in discharge is comparable to that modeled by Kunstmann et al. (2009) who estimated a 10-15% decrease in precipitation in the upper part of the Jordan basin in the 2035-2060 period, compared to the PRECIS modeled decrease in precipitation of 15-20% by 2040-2069.

S6.3 Socio-economic impacts

Bahrain, Cyprus, Israel, Jordan, Kuwait, the Palestinian territories, Qatar and Syria currently have less than 1,000 m^3^ water per capita available per year, the threshold generally used to define water scarcity. Population projections (UN Population Division 2009) combined with the projected internal water resources suggest that by mid-century water shortage will worsen in all countries which are already water scarce. Cyprus and Jordan will have their per capita IWR reduced by approximately two-thirds, to 340 and 38 m^3^/year, respectively. Such changes will necessitate major adaptation in the agricultural sectors of both countries, which currently account for 65-75% of water use (FAO 2009b). In Jordan water resources would fall below the 50 m^3^ per capita/year threshold identified as the minimum amount required for social and economic development (Chenoweth 2008), suggesting that significant desalination will be required to meet basic needs, albeit the costly nature of this solution given Jordan’s geography. Syria may also have its per capita water resources reduced by nearly half, and thus will also require major social and economic change, especially since nearly 90% of current water withdrawals are for agriculture and 25% of the crop land is irrigated (FAO 2009a).

Desalination costs have steadily declined over the last few decades, with recent contracts providing desalinated seawater at a relatively low cost of $US 0.53/m^3^ in Israel and $US 0.45/m^3^ in Singapore (Gleick et al. 2006). With internal water resources across the region declining by 45 km^3^/year in the 2040-2069 period and 53 km^3^/year in 2070-2099, at an estimated price of $0.50 per cubic meter, the cost of replacing the lost volume of IWR through desalination would be $23 billion/year in the 2040-2069 period and $26 billion/year in the 2070-2099 period (in current US$). This compares to the total GDP across the EMME in 2008 of about $1,67 trillion (UNDP 2010). This suggests that although the climate change effects on water resources will be very costly, they are potentially manageable, although some countries with comparatively weak economies may be affected rather strongly.

**S7 Energy demand**

Recently, several reports have addressed energy use in view of climate change in the Mediterranean and North Africa (MENA) region (e.g. Tourre et al. 2008; ESMAP 2009). One conclusion is that from 1990 to 2025 the emissions of CO_2_ in this region are doubling, related to the growing use of fossil fuels, which dominate the energy supply. Further, in the southern part of the region, being under fast development, the emissions are growing much more rapidly than in the north. Actually, the energy consumption is growing at a higher rate than in any other part of the world, reflecting the expansion of energy-intensive industries in the Gulf States and the emergent demand for electricity and transport from growing populations (ESMAP 2009). However, for fossil energy importing countries – outside the Gulf area – the expected rise in supply costs will be connected to social and economic risks.

The demand for energy in the built environment, i.e. private, commercial and public buildings, is directly related to climatic conditions. However, the relationship is not linear. Changes in energy consumption are to a large degree linked to the variability of ambient air temperatures, and the maximum energy demand is closely connected to extreme values of air temperature (maximum and minimum). Considering the expected strong temperature increases in the EMME region, this section addresses the consequences for energy requirements using the PRECIS climate projections. We focus on the mid-century period as decision making and investments in the energy sector have a typical planning perspective of several decades.

S7.1 Heating and cooling degree-days

To gain insight about the relationship between temperature and energy use we use the concept of degree-days, defined as the difference (in °C) of the diurnal mean temperature compared to a base temperature at which the energy consumption is at minimum. Consequently, the degree-day index would be positive in the summer and negative in the winter. However, rather than applying positive and negative values the following definitions are used; heating (HDD) and cooling degree days (CDD):

HDD_i_ = max(T* − T_i_,0)

CDD_i_ = max(T_i_ − T**,0)

where T* and T** are the base temperatures for HDD and CDD, respectively, and T_i_ is the mean temperature of day i.

The HDD_i_ and CDD_i_ values are typically cumulated over a specified period (annual or seasonal) to provide an indication of the severity of winter (summer) conditions at a particular location in terms of the outdoor dry-bulb air temperature, which in turn offers a guide to the likely aggregate energy demand for sensible heating (cooling) during that period. Beenstock et al. (1999) defined the HDD in Israel by taking T* = 10°C and the CDD by T** = 25°C. Within these two limits a comfort zone can be defined, in which no heating or cooling is required. Kadioğlu et al. (2001) used the different base levels 15° and 24°C for calculations of HDD and CDD in Turkey, respectively. Cartalis et al. (2001), in their study of the southeastern Mediterranean, used the threshold values of 15.5° and 18°C for HDD and CDD, respectively. To calculate CDDs for the warm season (June­-September) in Greece, base temperatures of 25° and 28°C were used (Tselepidaki et al. 1994). Here we use 15°C for the calculation of HDDs and 25°C for CDDs, as defined by Giannakopoulos et al. (2009a).

S7.2 Cooling energy requirements

The annual cumulative CDD in the EMME ranges widely, in the 1961-1990 reference period from zero to about 1,000, with the lowest values in the northern mountain ranges and highest in the southern deserts, especially around the Arabian Gulf. In the Mediterranean northern coastal regions they are typically less than 100 and in the southern coastal regions less than 200 CDD. Our PRECIS output-based calculations for 2040-2069 suggest that in the northern part of the domain (>36°-38°N) the CDDs will typically increase by 100-200, along the eastern and southern Mediterranean coasts by 200-300, in the deserts of Lybia, Egypt, Iraq and Saudi Arabia by 500-700, and in the southern Gulf states by up to 900. Most of these increases will occur in summer and some in the autumn, the latter around the Gulf (200-250 in September-November).

An illustrative and relevant view of the increasing cooling demands in the EMME is provided by the mean number of days per year during which cooling will need to exceed 5°C (CDD>5°C) by comparing the control and mid-century periods. This index indicates the additional strong cooling needed to provide comfortable living conditions and cope with heat waves. Fig. S7 shows that during the control period the CDD>5°C in the northern and coastal EMME is typically less than a few weeks to one month, while this is 2-3 months in the southern desert areas and up to 5 months around the Gulf. Further, the model predicts quite dramatic increases in CDD for the period 2040-2069, with 3-6 weeks in the northern and coastal areas, one month around the Gulf, up to 2 months in parts of Syria, southern Israel, Jordan, parts of Saudi Arabia, Egypt and Libya.

For comparison, Alcamo et al. (2007) and Giannakopoulos et al. (2009a) state that by the end-of-century along most of the northern Mediterranean coast an additional 2-3 weeks and further inland up to 5 more weeks of intense cooling will be needed. Moreover, the study of Giannakopoulos et al. (2009b), based on the ensemble output of 6 regional models, shows that in North Africa more than one additional month of heavy cooling will be required whereas in eastern Greece, western Turkey and Cyprus 15 additional days of heavy cooling will be needed. These findings underscore the relatively strong impact of climate change in the EMME.

The peak additional cooling energy demand during the warm and dry future summers coincides with a deficit in water supply, which reduces energy production by hydroelectric plants; for example in Turkey, where hydropower currently accounts for about 30% of the electricity production (Alcamo et al. 2007). In addition, these conditions will coincide with a growing demand for desalinated water. To put this into perspective, in Israel about 3% of national energy production is currently used for desalination, which may change approximately proportionally with the increasing water requirements. In EMME countries with limited resources, the anticipated increasing need for space cooling and fresh water demand will lead to a growing disparity in the power supply, and a growing need for innovative solutions such as the co-generation of electricity and desalinated seawater by using solar power.

S7.3 Heating energy requirements

Heating requirements in the EMME will also change especially in winter and to a lesser degree in spring, for example in Greece and Turkey. The decrease is most evident in the northern continental parts of the region, which reach about 90 HDD in winter and 60 in spring, by comparing the recent and mid-century periods. Given the high temperature conditions in the southern EMME, the heating requirements are minor. Nevertheless, small decreases in the winter cumulative HDD are projected in North Africa and the Middle East, whereas during other seasons the changes are insignificant. Again, it is useful to provide an additional perspective through changes of the mean annual HDD>5°C (extreme heating) between the two periods. Our calculations suggest that HDD>5°C will decrease by about 3-6 weeks in continental Greece, Turkey and Iran to about two weeks in Mediterranean islands such as Cyprus and less in the southern EMME, consistent with the results of Giannakopoulos et al (2009b). Even though changes in CDD and HDD to some degree compensate in terms of energy use and CO_2_ emissions, they do not occur in the same countries and will not help solve the growing problems with energy supply and cooling requirements during summer.

**References**

Abril JM, Abdel-Aal MM (2000) A modelling study on hydrodynamics and pollutant dispersion in the Suez Canal. Ecol Model 128: 1-17

Ainsworth EA, Leakey ADB, Ort DR, Long SP (2008) FACE-ing the facts: inconsistencies and interdependence among field, chamber and modeling studies of elevated CO_2_ impacts on crop yield and food supply. New Phytol 179: 5-9

Alcamo J et al. (2007) Europe. In: Parry ML et al. (eds) Climate Change 2007. Impacts, adaptation and vulnerability. Contribution of Working Group II to the Fourth Assessment Report of the Intergovernmental Panel on Climate Change (IPCC). Cambridge University Press, Cambridge, UK, pp 541-580

Alpert P et al. (2006) Relations between climate variability in the Mediterranean region and the Tropics: ENSO, South Asian and African Monsoons, Hurricanes and Saharan dust. In: Lionello P, Malanotte-Rizzoli P, Boscolo R (eds) Mediterranean climate variability. Elsevier, Amsterdam

Alten B, Selim Caglar S, Özer N (2000) Malaria and its vectors in Turkey. European Mosquito Bulletin 7: 27-33

Anderson GB, Bell ML (2011) Heat waves in the United States: Mortality risk during heat waves and effect modification by heat wave characteristics in 43 US communities. Environ Health Pers 119: 210-216

Artale V, Calmanti S, Carillo A, Dell'Aquila A, Herrmann M, Pisacane G, Ruti PM, Sannino G, Struglia MV, Giorgi F, Bi XQ, Pal JS, Rauscher S, Grp, P (2010) An atmosphere-ocean regional climate model for the Mediterranean area: assessment of a present climate simulation. Clim Dyn 35: 721-740

Bakun A, Agostini VN (2001) Seasonal patterns of wind-induced upwelling/downwelling in the Mediterranean Sea. Sci Marina 65: 243-257

Battisti DS, Naylor RL (2009) Historical warnings of future food insecurity with unprecedented seasonal heat. Science 323:240-244

Beenstock M, Goldin E, Nabot D (1999) The demand for electricity in Israel. Energy Econ 21: 168-183

Ben-Zvi A, Givati A (2008) Comment on First super-high-resolution model projection that the ancient "Fertile Cresent" will disappear in this century by Akio Kitoh, Akiyo Yatagai and Pinhas Alpert'. Hydrol Res Lett 2: 45

Béranger K, Mortier L, Crépon M (2005) Seasonal variability of water transport through the Straits of Gibraltar, Sicily and Corsica, derived from a high-resolution model of the Mediterranean circulation. Progr Ocean 66: 341-364

Bernabeu-Wittel M, Ruiz-Pérez M, del Toro MD, Aznar J, Muniain Á, de Ory F Domingo C, Pachón J (2007) West Nile virus past infections in the general population of Southern Spain. Enferm Infec Microbiol Clin 25: 561-565

Béthoux JP, Gentili B, Morin P, Nicolas E, Pierre C, Ruiz-Pino D (1999) The Mediterranean Sea: a miniature ocean for climatic and environmental studies and a key for the climatic functioning of the North Atlantic. Progr Ocean 44: 131-146

Beuvier J, Sevault F, Herrmann M, Kontoyannis H, Ludwig W, Rixen M, Stanev E, Beranger K, Somot S (2010) Modeling the Mediterranean Sea interannual variability during 1961–2000: focus on the Eastern Mediterranean Transient. J Geophys Res 115: C08017 doi:08010.01029/02009JC005950

Brasseur GP, Schultz M, Granier C, Saunois M, Diehl T, Botzet M, Roeckner E (2006) Impact of climate change on the future chemical composition of the global troposphere. J Climate 19: 3932-3951

Brönnimann S (2007) Impact of El Niño–Southern Oscillation on European climate. Rev Geophys 45: RG3003, doi:10.1029/2006RG000199

Brook RD, Rajagopalan S, Pope CA III, Brook JR, Bhatnagar A, Diez-Roux AV, Holguin F, Hong Y, Luepker RV, Mittleman MA, Peters A, Siscovick D, Smith SC Jr, Whitsel L, Kaufman JD on behalf of the American Heart Association Council on Epidemiology and Prevention, Council on the Kidney in Cardiovascular Disease, and Council on Nutrition, Physical Activity and Metabolism Circulation (2010) Particulate matter air pollution and cardiovascular disease: An update to the scientific statement from the American Heart Association. Circulation 121: 2331-2378

Burnett HS, Matthews M Jr (1997) Sick Argument: Global Warming and the Spread of Tropical Diseases. National Center for Policy Analysis, Washington DC

Butler TM, Lawrence MG, Gurjar BR, van Aardenne J, Schultz M, Lelieveld J (2008) The representation of emissions from megacities in global emission inventories. Atmos Environ 42: 703-719

Cartalis C, Synodinou A, Proedrou M, Tsangrassoulis A, Santamouris M (2001) Modifications in energy demand in urban areas as a result of climate changes: an assessment for the southeast Mediterranean region. Ener Conv. Manage. 2001, 42: 1647-1656

Chen CC, McCarl BA (2001) An investigation of the relationship between pesticide usage and climate change. Clim Change 50: 475-487

Chenoweth J (2008) Minimum water requirement for social and economic development. Desalination 229: 245-256

Chenoweth J, Hadjinicolaou P, Bruggeman A, Lelieveld J, Levin Z, Lange MA, Xoplaki E, Hadjikakou M (2011) The impact of climate change on the water resources of the eastern Mediterranean and Middle East region: modeled changes and socio-economic implications. Water Resour Res 47: W06506, doi: 10.1029/2010WR010269.

Chiew FHS (2006) Estimation of rainfall elasticity of streamflow in Australia. Hydrol Sci 51: 613-625

Ciais, P et al. (2005) Europe-wide reduction in primary productivity caused by the heat and drought in 2003. Nature 437: 529-533

CIESM (2008) Briand F (ed) Climate warming and related changes in Mediterranean marine biota. CIESM Workshop Monograph 35, Monaco

Coll M et al. (2010) The biodiversity of the Mediterranean Sea: estimates, patterns, and threats. Plos One 5: 10.1371/journal.pone.0011842

Collins WJ, Sitch S, Boucher O (2010) How vegetation impacts affect climate metrics for ozone precursors. J Geophys Res 115: D23308, doi: 10.1029/2010JD014187

Confalonieri U, Menne B, Akhtar R, Ebi KL, Hauengue M, Kovats RS, Revich B, Woodward A (2007) Human health. In: Parry ML et al. (eds) Climate Change 2007: Impacts, Adaptation and Vulnerability. Contribution of Working Group II to the Fourth Assessment Report of the Intergovernmental Panel on Climate Change. Cambridge University Press, Cambridge, UK, pp 391-431

Cowling RM, Rundel PW, Lamont BB, Arroyo MK, Arianoutsou M (1996) Plant diversity in Mediterranean climate regions. Trends Ecol Evol 11: 362-366

Cullen H M, deMenocal PB (2000) North Atlantic influence on Tigris–Euphrates streamflow*.* Int J Climatol 20: 853-863

Dallman PR (1998) Plant Life in the World’s Mediterranean Climates. University of California Press, Berkeley and Los Angeles, CA

de Dios VR, Fischer, C, Colinas C (2007) Climate change effects on Mediterranean forests and preventive measures. New Forests: 3329-3340

de Meij A, Pozzer A, Lelieveld J (2011) Global and regional trends in aerosol optical depth based on remote sensing data and pollutant emission estimates between 2000 and 2009. Atmos Chem Phys Discuss 10: 30731-30776

Diaz-Delgado R, Lloret F, Pons X, Terradas J (2002) Satellite evidence of decreasing resilience in Mediterranean plant communities after recurrent wildfires. Ecology 83: 2293-2303

D’Ippoliti, D et al. (2010) The impact of heat waves on mortality in 9 European cities: results from the EuroHEAT project. Environ Health 9: 37

Dimitrakopoulos AP, Vlahou M, Anagnostopoulou CG, Mitsopoulos ID (2011) Impact of drought on wildland fires in Greece: Implications of climate change? Clim Change, doi: 10.1007/s10584-011-0026-8

Doudier B, Bogreau H, DeVries A, Ponçon N, Stauffer W, Fontenille D, Rogier C, Parola P (2007) Possible autochthonous malaria from Marseille to Minnesota. Emerg Infect Dis 13: 1236-1238

Dujardin J-C, Campino L, Cañavate C, Dedet J-P, Gradoni L, Soteriadou K, Mazeris A, Ozbel Y, Boelaert M (2008) Spread of vector-borne diseases and neglect of Leishmaniasis, Europe. Emerg Infect Dis 14: 1013-1018

Dünkeloh A, Jacobeit , J (2003) Circulation dynamics of Mediterranean precipitation variability 1948–98. Int J Climatol 23: 1843-1866

Ebi KL, Paulson J (2007) Climate change and children. Pediatr Clin North Am 54: 213-226

El Maayar M, Ramankutty N, Kucharik C (2006) Modelling global and regional net primary production under elevated atmospheric CO_2_: On a potential source of uncertainty. Earth Interact 10: 1-20

El Maayar, M, Sonnentag O (2009) Crop model validation and sensitivity to climate change scenarios. Clim Res 39: 47-59

Energy Sector Management Assistance Program, ESMAP (2009) Tapping a hidden resource: Energy efficiency in the Middle East and North Africa, World Bank, Washington, DC

EPISOUTH (2008) Epidemiology of Crimean-Congo haemorrhagic fever virus: Albania, Bulgaria, Greece, Islamic Republic of Iran, Kosovo, Russian Federation, Turkey. 1st October 2008

Epstein PR (2001) West Nile Virus and the climate. J Urban Health 78: 367-371

Estrada-Peña A, Venzal JM (2007) Climate niches of tick species in the Mediterranean region: modeling of occurrence data, distributional constraints, and impact of climate change. J Med Entomol 44: 1130-1138

European Commission Joint Research Centre, ECJRC (2004) The Land Cover of the World in the Year 2000, http://bioval.jrc.ec.europa.eu/products/glc2000/glc2000.php

Evans JP (2009) 21st century climate change in the Middle East. Clim Change 92: 417-432

Evans, JP (2010) Global warming impact on the dominant precipitation processes in the Middle East. Theor Appl Climatol 99: 389-402

Fakeeh M, Zaki AM (2003) Dengue in Jeddah, Saudi Arabia, 1994-2002. WHO Dengue Bulletin 27:13-18

Feidas H, Noulopoulou C, Makrogiannis T, Bora-Senta E (2007) Trend analysis of precipitation time series in Greece and their relationship with circulation using surface and satellite data: 1955–2001. Theor Appl Climatol, 87: 155-177

Feki I, Marrakchi C, Ben Hmida M, Belahsen F, Ben Jemaa M, Maaloul I, Kanoun F, Ben Hamed S, Mhiri C (2005) Epidemic West Nile virus encephalitis in Tunisia. Neuroepidemiology 24: 1-7

Feliks Y, Ghil M, Robertson AW (2010) Oscillatory Climate Modes in the Eastern Mediterranean and Their Synchronization with the North Atlantic Oscillation. J Clim 23: 4060-4079

Felis T, Rimbu N (2010) Mediterranean climate variability documented in oxygen isotope records from northern Red Sea corals - A review. Glob Planet Change 71: 232-241

Fischer EM, Schär C (2010) Consistent geographical patterns of changes in high-impact European heatwaves. Nature Geosci 3: 398-403

Food and Agriculture Organization (FAO) Land and Water Development Division (2009a) Irrigation in the Middle East region in Figures: AQUASTAT survey – 2008. Food and Agriculture Organisation, Rome

Food and Agriculture Organization (FAO) Land and Water Development Division (2009b) Aquastat: FAO's Information System on Water and Agriculture. Food and Agriculture Organization, Rome

Formenti P, Andreae MO, Andreae T, Galani E, Vasaras A, Zerefos C, Amiridis V, Orlovsky L, Karnieli A, Wendisch M, Wex H, Holben BN, Maenhaut W, Lelieveld J (2001) Aerosol optical properties and large-scale transport of air masses: Observations at a coastal and semi-arid site in the eastern Mediterranean during summer 1998. J Geophys Res 106: 9807-9826

Gale P, Estrada-Peña A, Martinez M, Ulrich RG, Wilson A, Capelli G, Phipps P, de la Torre A, Muñoz MJ, Dottori M, Mioulet V, Fooks AR (2008) The feasibility of developing a risk assessment for the impact of climate change on the emergence of Crimean-Congo haemorrhagic fever in livestock in Europe: a review. J Appl Microbiol 108: 1859-1870

Ganor E, Osetinsky I, Stupp A, Alpert P (2010) Increasing trend of African dust, over 49 years, in the eastern Mediterranean. J Geophys Res 115: D07201, doi:10.1029/2009JD012500

Ganzeveld L, Bouwman L, Stehfest E, van Vuuren DP, Eickhout B, Lelieveld J (2010) The impact of future land-use and land-cover changes on atmospheric chemistry-climate interactions. J Geophys Res 115: D23301, doi:10.1029/2010JD014041

Gao X, Giorgi, F (2008) Increased aridity in the Mediterranean region under greenhouse forcing estimated from high resolution simulations with a regional climate model. Glob Planet Change 62: 195-209

García-Romero, A, Muñoz J, Andrés N, Palacios D (2010) Relationship between climate change and vegetation distribution in the Mediterranean mountains: Manzanares Head valley, Sierra De Guadarrama (Central Spain). Clim Change 100: 645-666

Giannakopoulos C, Le Sager P, Bindi M, Moriondo M, Kostopoulou E, Goodess CM (2009a) Climatic changes and associated impacts in the Mediterranean resulting from a 2°C global warming. Global Planet Change 68: 209-224

Giannakopoulos C, Hadjinicolaou P, Zerefos C, Demosthenous G (2009b) Changing energy requirements in the Mediterranean under changing climatic conditions. Energies 2(4): 805-815

Gibelin A L, Déqué M (2003) Anthropogenic climate change over the Mediterranean region simulated by a global variable resolution model. Clim Dyn 20: 327-339

Gleick PH, Cooley H, Wolff G (2006) With a grain of salt: an update on seawater desalination. In: Gleick PH (ed) The World's Water 2006-2007: The biennial report on freshwater resources. Island Press, Washington DC, pp. 51-90

Good P, Moriondo M, Giannakopoulos C, Bindi M (2008) The meteorological conditions associated with extreme fire risk in Italy and Greece: relevance to climate model studies. Int J Wildland Fire 17: 155-165

Guerova G, Jones N (2007) A global model study of ozone enhancement during the August 2003 heat wave in Europe. Environ Chem 4: 285-292

Gurjar BR, Jain A, Sharma A, Agarwal A, Gupta P, Nagpure AS, Lelieveld J (2010) Human health risks in megacities due to air pollution. Atmos Environ 44: 4606-4613

Haines A, Kovats RS, Campbell-Lendrum D, Corvalan C (2006) Climate change and human health: impacts, vulnerability, and mitigation. Lancet 367: 2101-2109

Hamad N, Millot C, Taupier-Letage I (2006) The surface circulation in the eastern basin of the Mediterranean Sea. Sci Marina 70: 457-503

Hartley LM, Donnelly CA, Garnett GP (2002) The seasonal pattern of dengue in endemic areas: mathematical models of mechanisms. Trans R Soc Trop Med Hyg 96: 387-397

Hasanean HM (2004) Variability of the North Atlantic subtropical high and associations with tropical sea-surface temperature*.* Int J Climatol 24: 945-957

Hegazy AK, Medany MA, Kabiel HF, Maez MM (2008) Spatial and temporal projected distribution of four crop plants in Egypt. Nat Res Forum 32: 316-326

Held IM, Soden BJ (2006) Robust responses of the hydrological cycle to global warming. J Clim 19: 5686-5699

Henkin Z, Perevolotsky A, Sternberg M (2010) The contributions of grasslands to the conservation of Mediterranean biodiversity. Opt Méditerran A 92: 167-174

Hertel TW, Burke MB, Lobell DB (2010) The poverty implications of climate-induced crop yield changes by 2030. Glob Environ Change 20: 577-585

Hildebrandt L, Engelhart GJ, Mohr C, Kostenidou E, Lanz VA, Bougiatioti A, DeCarlo PF, Prevot ASH, Baltensperger U, Mihalopoulos N, Donahue NM, Pandis SN (2010) Aged organic aerosol in the Eastern Mediterranean: the Finokalia Aerosol Measurement Experiment – 2008. Atmos Chem Phys 10: 4167-4186

Hirschi, M, Seneviratne SI, Alexandrov V, Boberg F, Boroneant C, Christensen OB, Formayer H, Orlowski B, Stepanek P (2011) Observational evidence for soil-moisture impact on hot extremes in southeastern Europe. Nature Geosci 4: 17-21

Hoekstra JM, Boucher TM, Ricketts TH, Roberts C (2005) Confronting a Ziome crisis: global disparities of habitat loss and protection. Ecol Lett 8: 23-29

Holland M, Kinghorn S, Emberson L, Cinderby S, Ashmore M, Mills G, Harmens H (2006) Development of a framework for a probabilistic assessment of the economic losses caused by ozone damage to crops in Europe. Centre for Ecology and Hydrology, Bangor, UK

Huang J, Higuchi K, Shabbar A (1998) The relationship between the North Atlantic Oscillation and El Niño–Southern Oscillation. Geophys Res Lett 25: 2707-2710

Hurrell JW (2003) Climate Variability: North Atlantic and Arctic Oscillation. In: Holton J, Pyle J, Curry J (eds) Encyclopedia of Atmospheric Sciences. Academic Press, Amsterdam, pp 439-445

Huston MA (1994) Biological diversity: the coexistence of species on changing landscapes. Cambridge University Press, Cambridge, UK

IPCC (2007) Climate Change 2007: The Physical Science Basis. Contribution of Working Group I to the Fourth Assessment Report of the Intergovernmental Panel on Climate Change, Solomon S et al. (eds), Cambridge University Press, Cambridge, UK

Jaffe CL, Baneth G, Abdeen ZA, Schlein Y, Warburg A (2004) Leishmaniasis in Israel and the Palestinian Authority. Trends Parasitol 20: 328-332

Kadioğlu M, Tulunay Y, Borhan Y (1999) Variability of Turkish precipitation compared to El Niño events. Geophys Res Lett 26: 1597-1600

Kadioğlu M, Şen Z, Gultekin L (2001) Variations and trends in Turkish seasonal heating and cooling degree-days. Clim Change 49: 209-223

Kallos G, Kotroni V, Lagouvardos K, Papadopoulos A (1998) On the long-range transport of air pollutants from Europe to Africa. Geophys Res Lett 25: 619-622

Karl DM, Björkman KM, Dore JE, Fujieki L, Hebel DV, Houlihan T, Letelier R, Tupas LM (2001) Ecological nitrogen-to-phosphorus stoichiometry at station ALOHA. Deep Sea Res II 48: 1529-1566

Katsouyanni K, Trichopoulos D, Zavitsanos X, Touloumi G (1988) The 1987 Athens heat wave. The Lancet 332: 8610, 573

Katsouyanni K, Pantazopoulou A, Touloumi G (1993) Evidence for interaction between air pollution and high temperature in the causation of excess mortality. Arch Environ Health 48: 235-242

Kitoh A, Yatagai A, Alpert P (2008) First high-resolution model projection that the ancient “Fertile Crescent” will disappear in this century. Hydrol Res Lett 2: 1-4

Klausmeyer KR, Shaw MR (2009) Climate change, habitat loss, protected areas and the climate adaptation potential of species in the Mediterranean ecosystems worldwide. Plos One 4(7): 1-9

Klein B, Roether W, Kress N, Manca BB, d'Alcala MR, Souvermezoglou E, Theocharis A, Civitarese G, Luchetta A (2003) Accelerated oxygen consumption in eastern Mediterranean deep waters following the recent changes in thermohaline circulation. J Geophys Res 108: 8107, doi:10.1029/2002JC001454

Knowlton K, Rotkin-Ellman M, King G, Margolis HG, Smith D, Solomon G, Trent R, English P (2009) The 2006 California heat wave: Impacts on hospitalizations and emergency department visits. Environ Health Pers 117: 61-67

Kopel E, Schwartz E, Amitai1 Z, Volovik I (2010) Relapsing vivax malaria in Eritrean refugees, refugees, Israel, June 2010. Euro Surveill 15(26): 19601

Koster RD, Suarez MJ, Higgins RW, Van den Dool HM (2003) Observational evidence that soil moisture variations affect precipitation. Geophys Res Lett 30: 1241, doi: 10.1029/2002GL016571

Kouvarakis G, Doukelis Y, Mihalopoulos N, Rapsomanikis S, Sciare J, Blumthaler M (2002) Chemical, physical and optical characterization of aerosol during the PAUR II experiment. J Geophys Res 107: 8141, doi:10.1029/2000JD000291

Kress N, Manca BB, Klein B, Deponte D (2003) Continuing influence of the changed thermohaline circulation in the eastern Mediterranean on the distribution of dissolved oxygen and nutrients: Physical and chemical characterization of the water masses. J Geophys Res 108: 8109, doi:10.1029/2002JC001397

Krichak SO, Alpert P (2005) Decadal trends in the east Atlantic–west Russia pattern and Mediterranean precipitation. Int J Climatol 25: 183-192

Krom MD, Woodward EMS, Herut B, Kress N, Carbo P, Mantoura RFC, Spyres G, Thingstad TF, Wassman P, Wexels-Riser C, Kitidis V, Law CS, Zodiatis G (2005) Nutrient cycling in the south east Levantine basin of the eastern Mediterranean: Results from a phosphorus starved system. Deep Sea Res II 52, 2879-2896

Kuglitsch FG, Toreti A, Xoplaki E, Della Marta PM, Zerefos CS, Türkeş M, Luterbacher J (2010) Heat wave changes in the eastern Mediterranean since 1960. Geophys Res Lett 37: L04802, doi:10.1029/2009GL041841

Kunstmann H, Heckl A, Suppan P, Smiatek G, Alpert P, Krichak S, Samuels R, Breitgand J, Jin F (2009) P3: Regional Climate Scenarios, An integrated approach to sustainable management of water resources under global change. GLOWA, Jordan River

Kutiel H, Maheras P, Türkeş M, Paz S (2002) North Sea – Caspian Pattern (NCP) – an upper level atmospheric teleconnection affecting the eastern Mediterranean – implications on the regional climate. Theor Appl Climatol 72: 173-192

Lascaratos A, Nittis K (1998) A high-resolution three-dimensional numerical study of intermediate water formation in the Levantine Sea. J Geophys Res 103: 18497-18511

Leff B, Ramankutty N, Foley J (2004) Geographic distribution of major crops across the world. Glob Biogeo Cycles 18: GB1009, doi:10.1029/2003GB002108

Le Houérou HN (2002) Man-made deserts: Desertizatization processes and threats. Arid Land Res Managem 16: 1-36

Lelieveld J, Berresheim H, Borrmann S, Crutzen PJ, Dentener FJ et al. (2002) Global air pollution crossroads over the Mediterranean. Science 298: 794-799

Lelieveld J (2009) Air pollution and climate. In: Woodward JC (ed) The physical geography of the Mediterranean, Oxford University Press, Oxford, UK, pp. 599-614

Lelieveld J, Hoor, P, Jöckel P, Pozzer A, Hadjinicolaou P, Cammas JP (2009) Severe ozone air pollution in the Persian Gulf region. Atmos Chem Phys 9: 1393-1406

Lindgren E, Naucke T, Menne B (2004) Climate variability and visceral Leishmaniasis in Europe. In: Report of the Scientific Working Group meeting on Leishmaniasis, 2-4 February 2004, Geneva: 88-93

Lionello P, Malanotte-Rizzoli P, Boscolo R (eds) (2006) Mediterranean Climate Variability. Elsevier, Amsterdam

Lobell DB, Burke MB (2008) [Why are agricultural impacts of climate change so uncertain? The importance of temperature relative to precipitation.](http://iopscience.iop.org/1748-9326/3/3/034007)  Environ Res Lett 3: 034007 doi: [10.1088/1748-9326/3/3/034007](http://dx.doi.org/10.1088/1748-9326/3/3/034007)

Long SP, Ainsworth EA, Leakey ADB, Morgan PB (2009) Global food insecurity. Treatment of major food crops with elevated carbon dioxide or ozone under large-scale fully open-air conditions suggests recent models may have overestimated future yields. Phil Trans R Soc B, 360: 2011-2020

Malanotte-Rizzoli P, Manca BB, D'Alcala MR, Theocharis A, Bergamasco A, Bregant D, Budillon G, Civitarese G, Georgopoulos D, Michelato A, Sansone E, Scarazzato P, Souvermezoglou E (1997) A synthesis of the Ionian Sea hydrography, circulation and water mass pathways during POEM Phase I. Prog Ocean 39: 153-204

Malanotte-Rizzoli P et al. (2003) The Levantine Intermediate Water Experiment (LIWEX) Group: Levantine basin - A laboratory for multiple water mass formation processes. J Geophys Res 108: 8101, doi:10.1029/2002JC001643

Manca B, Burca M, Giorgetti A, Coatanoan C, Garcia MJ, Iona A (2004) Physical and biochemical averaged vertical profiles in the Mediterranean regions: an important tool to trace the climatology of water masses and to validate incoming data from operational oceanography. J Marine Sys 48: 83-116

Marcos M, Tsimplis MN (2008) Comparison of results of AOGCMs in the Mediterranean Sea during the 21st century. J Geophys Res 113: C12028, doi:10.1029/2008JC004820

Mariotti A, Ning Z, Yoon JH, Artale V, Navarra A, Alpert P, Li LZX (2008) Mediterranean water cycle changes: transition to dryer 21^st^ century conditions in observations and CMIP3 simulations. Environ Res Lett 3: 044001

Marmer E, Langmann B (2005) Impact of ship emissions on Mediterranean summertime pollution and climate: A regional modeling study. Amos Environ 39: 4659-4669

Marr JS, Calisher CH (2003) Alexander the Great and West Nile virus encephalitis. Emerg Infect Dis 9: 1599-1603

McMichael AJ (2003) Global climate change and health: an old story writ large. In: McMichael AJ, Campbell-Lendrum DH, Corvalán CF et al. (eds) Climate change and human health – risks and responses. WHO Geneva, pp 1-17

McMichael AJ, Woodruff RE, Hales S (2006) Climate change and human health: present and future risks. The Lancet 367: 859-869

Mihalopoulos N, Stephanou E, Kanakidou M, Pilitsidis S, Bousquet P (1997) Tropospheric aerosol ionic composition above the Eastern Mediterranean Area. Tellus 49B: 314-326

Millán MM, Mantilla E, Salvador R, Carratalá R, Sanz MJ, Alonso L, Gangioti G, Navazo M (2000) Ozone cycles in the western Mediterranean basin: Interpretation of monitoring data in complex coastal terrain. J Appl Meteor 39: 487-508

Millán, MM, Estrela MJ, Sanz MJ, Mantilla E, Martin M, Pastor F, Salvador R, Vallejo R, AlonsoL, Gangioti G, Ilardia LJ, Navazo M, Albizuri A, Artiñano B, Ciccioli P, Kallos G, Carvalho RA, Andrés D, Hoff A, Werhahn J, Seufert G, Versino B (2005) Climate feedbacks and desertification: The Mediterranean model. J Climate 18: 684-701

Millot C, Candela J, Fuda JL, Tber Y (2006) Large warming and salinification of the Mediterranean outflow due to changes in its composition. Deep Sea Res I 53: 656-666

Montoya, R (1995) Red de seguimiento de danos en los montes. Danos originados por la sequıa en 1994. Cuadernos de la Sociedad Espanola de Ciencias Forestales 2 : 83-97

Moriondo M, Good P, Durao R, Bindi M, Giannakopoulos C, Corte-Real J (2006) Potential impact of climate change on fire risk in the Mediterranean area. Clim Res 31: 85-95

Moriondo M, Stefanini FM, Bindi M (2008) Reproduction of olive tree habitat suitability for global change impact assessment. Ecol Model 218: 95-109

Moriondo M, Giannakopoulos C, Bindi M (2010) Climate change impact assessment: the role of climate extremes in crop yield simulation. Clim Change 104: 679-701

Muñoz-Díaz D, Rodrigo FS (2005) Influence of the El Niño–Southern Oscillation on the probability of dry and wet seasons in Spain. Clim Res 30: 1-12

Murakami M (1995) Managing water for peace in the Middle East: Alternative Strategies. United Nations University Press, Tokyo

Nelson GC, Rosegrant MW, Koo J, Robertson R, Sulser T, Zhu T, Ringler C, Msangi S, Palazzo A, Batka M, Magalhaes M, Valmonte-Santos R, Ewing M, Lee D (2009) Climate Change: Impact on Agriculture and Costs of Adaptation. IFPRI, Food Policy Report

Nissen KM, Leckebusch GC, Pinto JG, Renggli D Ulbrich S, Ulbrich U (2010) Cyclones causing wind storms in the Mediterranean: characteristics, trends and links to large-scale patterns. Nat Hazards Earth Syst Sci 10: 1379-1391

Nykjaer L (2009) Mediterranean Sea surface warming 1985-2006. Clim Res 39: 11-17

Olson, DM, Dinerstein E (2002) The Global 200: Priority ecoregions for global conservation. Ann Mo Bot Gard 89: 199-224

Özsoy E, Hecht A, Ünlüata U, Brenner S, Sur HI, Bishop J, Latif MA, Rozentraub Z, Oguz, T (1993) A synthesis of the Levantine Basin circulation and hydrography, 1985-1990. Deep Sea Res II 40: 1075-1119

Pacsa A, Mustafa AS, Chaturvedi UC (2002) Study of Dengue Virus Infection in Kuwait. WHO Dengue Bull 26: 113-117

Papadimas CD, Hatzianastassiou N, Mihalopoulos N, Kanakidou M, Katsoulis BD, Vardavas I (2009) Assessment of the MODIS Collections C005 and C004 aerosol optical depth products over the Mediterranean basin. Atmos Chem Phys 9: 2987-2999

Patz JA, Olson SH, Uejio CK, Gibbs HK (2008) Disease emergence from global climate and land use change. Med Clin N Am 92: 1473-1491

Paz S (2006) The West Nile Virus outbreak in Israel from a new perspective: The regional impact of climate change. Int J Env Health Res 16: 1-13

Paz S, Albersheim I (2008) Influence of Warming Tendency on Culex pipiens Population Abundance and on the Probability of West Nile Fever Outbreaks (Israeli Case: 2001-2005). EcoHealth 5: 40-48

Pinto JG, Zacharias S, Fink AH, Leckebusch GC, Ulbrich U (2009) Factors contributing to the development of extreme North Atlantic cyclones and their relationship with the NAO. Clim Dyn 32: 711-737

Ponçon N, Balenghien T, Toty C, Ferré JB, Thomas C, Dervieux A, L'Ambert G, Schaffner F, Bardin O, Fontenille D (2007) Effects of local anthropogenic changes on potential malaria vector Anopheles hyrcanus and West Nile virus vector Culex modestus, Camargue, France. Emerg Infect Dis: http://www.cdc.gov/EID/content/13/12/1810.htm

Pongsumpun P, Garcia Lopez D, Favier C, Torres L, Llosa J, Dubois MA (2008) Dynamics of dengue epidemics in urban contexts. Trop Med Int Health 13: 1180-1187

Pringle KJ, Tost, H, Metzger S, Steil B, Giannadaki D, Nenes A, Fountoukis C, Stier P, Vignati E, Lelieveld J (2010) Description and evaluation of GMXe: A new aerosol submodel for global simulations (v1). Geosci Model Develop 3: 391-412

Qian B, Corte-Real,J, Xu H (2000) Is the North Atlantic Oscillation the most important atmospheric pattern for precipitation in Europe? J Geophys Res 105: 11,901-11,910

Raes F, Liao H, Chen WT, Seinfeld JH (2010) Atmospheric chemistry‐climate feedbacks. J Geophys Res 115: D12121, doi:10.1029/2009JD013300

Raible CC (2007) On the relation between extremes of midlatitude cyclones and the atmospheric circulation using ERA40. Geophys Res Lett 34, L07703, doi:10.1029/2006GL029084

Rathor HR (2000) The role of vectors in emerging and re-emerging diseases in the eastern Mediterranean region. WHO Dengue Bull 24: 103-110

Ready PD (2010) Leishmaniasis emergence n Europe. Euro Surveill 15(10): 19505

Reisen WK, Thiemann T, Barker CM, Lu H, Carroll B, Fang Y, Lothrop HD (2010) Effects of warm winter temperature on the abundance and gonotrophic activity of Culex (Diptera: Culicidae) in California. J Med Entomol 47: 230-237

Reiter P (2001) Climate Change and Mosquito Borne Diseases. Environ Health Perspect 109: 141-161

Reiter P (2010) West Nile virus in Europe: understanding the present to gauge the future. Euro Surveill 15(10): 19508

Riera P, Penuelas J, Farrera V, Estiarte M (2007) Valuations of climate-change effects on Mediterranean shrublands. Ecol Appl 17: 91-100

Robine JM, Cheung SLK, Le Roy S, Van Oyen H, Griffiths C, Michel JP, Herrmann FR (2006) Death toll exceeded 70,000 in Europe during the summer of 2003. C R Biologies 331: 171-178

Robinson AR, Golnaraghi M, Leslie WG, Artegiani A, Hecht A, Lazzoni E, Michelato A, Sansone E, Theocharis A, Ünlüata Ü (1991) The eastern Mediterranean general circulation: features, structure and variability. Dyn Atmos Oceans 15: 215-240

Rodwell MJ, Hoskins BJ (1996). Monsoons and the dynamics of deserts Q J R Meteorol Soc 122: 1385-1404

Roether W, Manca BB, Klein B, Bregant D, Georgopoulos D, Beitzel V, Kovacevic V, Luchetta, A (1996) Recent changes in eastern Mediterranean deep waters. Science 271: 333-335

Roether W, Klein B, Manca BB, Theocharis A, Kioroglou S (2007) Transient Eastern Mediterranean deep waters in response to the massive dense-water output of the Aegean Sea in the 1990s. Prog Ocean 74: 540-571

Rohling EJ, Bryden HL (1992) Man-induced salinity and temperature increases in Western Mediterrnean Deep Water. J Geophys Res 97: 11191-11198

Romanou A, Tselioudis G, Zerefos CS, Clayson CA, Curry JA, Andersson A (2010) Evaporation-Precipitation variability over the Mediterranean and the Black Seas from satellite and reanalysis estimates. J Climate 23: 5268-5287

Rosenzweig C, Iglesias A, Yang XB, Epstein PR, Chivian E (2002) Climate change and extreme weather events: implications for food production, plant diseases, and pests. Glob Ch Hum Health 2: 90-104

Rowell DP, Jones RG (2006) Causes and uncertainty of future summer drying over Europe. Clim Dyn 27: 281-299

Sala OE, Chapin FS, Armesto JJ, Berlow E, Bloomfield J et al. (2000) Biodiversity - Global biodiversity scenarios for the year 2100. Science 287: 1770-1774

Sardans J, Peñuelas J, Ogaya R (2008) Experimental drought reduced acid and alkaline phosphatase activity and increased organic extractable P in soil in a Quercus ilex Mediterranean forest. Eur J Soil Biol 44: 509-520

Schär C, Lüthi D, Beyerle U, Heise E (1999) The soil–precipitation feedback: A process study with a regional climate model. J Climate 12: 722-741

Schwartz E (2005) Malaria – A disease that refuses to die but continues to kill. Isr Med Assoc J 7: 404-405.

Sciare J, Bardouki H, Moulin C, Mihalopoulos N (2003) Aerosol sources and their contribution to the chemical composition of aerosols in the Eastern Mediterranean Sea during summertime. Atmos Chem Phys 3: 291-302

Semenza JC, Menne B (2009) Climate change and infectious diseases in Europe. Lancet Infect Dis 9: 365-75

Siokou-Frangou I, Gotsis-Skretas O, Christou ED, Pagou K (1999) Plankton characteristics in the Aegean, Ionian and NW Levantine Seas. In: Malanotte-Rizzoli P (ed) The eastern Mediterranean as a laboratory basin for the assessment of contrasting ecosystems. Kluwer Academic Publishers, Dordrecht, 99 205-223

Siokou-Frangou I, Christaki U, Mazzocchi MG, Montresor M, d'Alcala MR, Vaque D, Zingone, A (2010) Plankton in the open Mediterranean Sea: a review. Biogeosci 7: 1543-1586

Sitch S, Cox PM, Collins WJ, Huntingford C (2007) Indirect radiative forcing of climate change through ozone effects on the land-carbon sink. Nature 448: 791-795

Skliris N, Sofianos S, Lascaratos A (2007) Hydrological changes in the Mediterranean Sea in relation to changes in the freshwater budget: A numerical modelling study. J Marine Sys 65: 400-416

Somot S, Sevault F, Deque M (2006) Transient climate change scenario simulation of the Mediterranean Sea for the twenty-first century using a high-resolution ocean circulation model. Clim Dyn 27: 851-879

Somot S, Sevault F, Deque M, Crepon, M (2008) 21st century climate change scenario for the Mediterranean using a coupled atmosphere-ocean regional climate model. Glob Planet Change 63: 112-126

Stavrakou T, Müller JF, Boersma KF, De Smedt I, van der A RJ (2008) Assessing the distribution and growth rates of NOx emission sources by inverting a 10‐year record of NO2 satellite columns. Geophys Res Lett 35: L10801, doi:10.1029/2008GL033521

Straetemans M, Almeida AP, Bellini R, Coulombier D, Depoortere E, Eritja R, Fontenille D, Giesecke J, Lundström LA, Medlock J, Merdic E, Payne L, Powers A, Romi R, Samanidou A, Schaffner F, Scholte EJ, Versteirt V (2008) Vector-related risk mapping of the introduction and establishment of Aedes albopictus in Europe. Euro Surveill 13: 8040

Struglia MV, Mariotti A, Filograsso, A (2004) River Discharge into the Mediterranean Sea: Climatology and Aspects of the Observed Variability. J Clim 17: 4740-4751

Tegen I, Werner M, Harrison SP, Kohfeld KE (2004) Relative importance of climate and land use in determining present and future global soil dust emission. Geophys Res Lett 31: L05105, doi:10.1029/2003GL019216

The Royal Society (2008) Ground-level ozone in the 21st century: future trends, impacts and policy implications. RS Policy document 15/08. ISBN: 978085403-7131

Thompson DWJ, Wallace JM (1998) The Arctic Oscillation signature in the wintertime geopotential height and temperature fields. Geophys Res Lett 25: 1297-1300

Thuiller W, Lavergne S, Roquet C, Boulangeat I, Lafourcade B, Aroujo MB (2011) Consequences of climate change on the tree of life in Europe. Nature 470: 531-534

Tilston N, Skelly C, Weinstein P (2009) Pan-European Chikungunya surveillance: designing risk stratified surveillance zones. Int J Health Geograph 8: 61

Tourre Y et al. (2008) Climate change and energy in the Mediterranean. Plan Bleu, Sophia Antipolis, Paris

Trigo RM, Pozo-Vázquez D, Osborn TJ, Castro-Díez Y, Gámis-Fortis S, Esteban-Parra M J (2004) North Atlantic Oscillation influence on precipitation, river flow and water resources in the Iberian Peninsula. Int J Climatol 24: 925-944

Trigo R et al. (2006) Relations between variability in the Mediterranean region and Mid-latitude variability. In: Lionello P, Malanotte-Rizzoli P, Boscolo R (eds) The Mediterranean Climate: an overview of the main characteristics and issues*.* Elsevier, Amsterdam, pp 179-226

Tselepidaki I, Santamouris M, Asimakopoulos DN, Kontoyiannidis S (1994) On the variability of cooling degree-days in an urban environment: application to Athens, Greece. Energy Bldg 21: 93-99

Tsimplis MN, Baker TF (2000) Sea level drop in the Mediterranean Sea: An indicator of deep water salinity and temperature changes?. Geophys Res Lett 27: 1731-1734

Tsimplis MN, Marcos M, Somot S. (2008) 21^st^ century Mediterranean sea level rise: Steric and atmospheric pressure contributions from a regional model. Glob Planet Change 63: 105-111

Tyrlis E, Hoskins BJ (2008a) The morphology of Northern Hemisphere blocking. J Atmos Sci 65: 1653-1665

Tyrlis E, Hoskins BJ (2008b) Aspects of Northern Hemisphere atmospheric blocking climatology. J Atmos Sci 65: 1638-1652

Tzali M, Sofianos S, Mantziafou A, Skliris N (2010) Modelling the impact of Black Sea water inflow on the North Aegean Sea hydrodynamics. Ocean Dyn 60: 585-596

Uherek E, Halenka T, Borken-Kleefeld J, Balkanski Y, Berntsen T, Borrego C, Gauss M, Hoor P, Juda-Rezler K, Lelieveld J, Melas D, Rypdal K, Schmid S (2010) Transport impacts on atmosphere and climate: Land transport. Atmos Environ 44: 4772-4816

United Nations Population Division (2009) World Population Prospects, the 2008 Revision. United Nations, New York

United Nations Development Program (UNDP) (2010) Human Development Report 2010. Oxford University Press, New York

Vandentorren S, Bretin P, Zeghnoun A, Mandereau-Bruno L, Croisier A, Cochet C, Riberon J, Siberan I, Declercq B, Ledrans M (2006) August 2003 heat wave in France: Risk factors for death of elderly people living at home. Eur J Publ Health 16: 583-591

van der A RJ, Eskes HJ, Boersma KF, van Noije TPC, van Roozendael M, De Smedt I, Peters DHMU, Meijer EW (2008) Trends, seasonal variability and dominant NOx source derived from a ten year record of NO_2_ measured from space. J Geophys Res 113: D04302, doi:10.1029/2007JD009021

Vargas-Yáñez M, Moya F, Garcia-Martinez MC, Tel E, Zunino P, Plaza F, Salat J, Pascual J, Lopez-Jurado JL, Serra M (2010a) Climate change in the Western Mediterranean Sea 1900-2008. J Marine Sys 82: 171-176

Vargas-Yáñez M, Zunino P, Benali A, Delpy M, Pastre F, Moya F, Garcia-Martinez MD, Tel E (2010b) How much is the western Mediterranean really warming and salting? J Geophys Res 115: C04001, doi:10.1029/2009JC005816

Vautard R, Yiou P, D’Andrea F, de Noblet N, Viovy N, Cassou C, Polcher J, Ciais P, Kageyama M, Fan Y (2007) Summertime European heat and drought waves induced by wintertime Mediterranean rainfall deficit. Geophys Res Lett 34: L07711, doi:10.1029/2006GL028001

Volk M, Bungener P, Contat F, Myrta M, Jurg F (2005) Grassland yield declined by a quarter in 5 years of free-air ozone fumigation. Glob Change Biol 12: 74-83

Vorou RM (2009) Crimean-Congo hemorrhagic fever in Southeastern Europe. Int J Infect Dis 13: 659-662

Wang C, Xie SP, Carton JA (2004) In: Wang C et al. (eds) A global survey of ocean atmosphere interaction and climate variability, in Earth’s Climate: The Ocean-Atmosphere Interaction. Geophys Monogr Ser 147. AGU, Washington, DC, pp 1-19

Wieliczko A, Staroniewicz Z (2010) Impact of global warming on the spread of infectious diseases. Medycyna Wet 66: 363-365

Wittig VE, Ainsworth EA, Long SP (2007) To what extent do current and projected increases in surface ozone affect photosynthesis and stomatal conductance of trees? A meta-analytic review of the last 3 decades of experiments. Plant Cell Environ 30: 1150-1162

World Health Organization (2006) WHO Air Quality Guidelines for Particulate Matter, Ozone, Nitrogen Dioxide and Sulfur Dioxide: Global Update 2005 (Summary of risk assessment) WHO/SDE/PHE/OEH/ 06.02, [http://whqlibdoc.who.int/hq/2006/ WHO_SDE_PHE_OEH 06.02_eng.pdf](http://whqlibdoc.who.int/hq/2006/%20WHO_SDE_PHE_OEH%2006.02_eng.pdf)

World Health Organization (WHO) Fact Sheet No. 94, April 2010 “MALARIA”. Available through: http://www.who.int/ mediacentre/factsheets/fs094/en/print.html

Xoplaki E (2002) Climate variability over the Mediterranean. Dissertation. University of Bern

Xoplaki E, González-Rouco JF, Luterbacher J, Wanner H (2004) Wet season Mediterranean precipitation variability: Influence of large-scale dynamics and trends. Clim Dynam 23: 63-78

Zavala JA, Casteel CL, DeLucia EH, Berenbaum MR (2008) Anthropogenic increase in carbon dioxide compromises plant defense against invasive insects. Proc Nat Acad Sc 105: 5129-5133

Zhang X et al. (2005) Trends in Middle East climate extreme indices from 1950 to 2003. J Geophys Res 110: D22104, doi:10.1029/2005JD006181

Zeng G, Pyle J (2003) Changes in tropospheric ozone between 2000 and 2100 modeled in a chemistry-climate model. Geophys Res Lett 30: 1392, doi:10.1029/2002GL016708

Zervoudaki S, Christou ED, Nielsen TG, Siokou-Frangou I, Assimakopoulou G, Giannakourou A, Maar M, Pagou K, Krasakopoulou E, Christaki U, Moraitou-Apostolopoulou M (2007) The importance of small-sized copepods in a frontal area of the Aegean Sea. J Plankton Res 29: 317-338

[Ziv](http://www.citeulike.org/user/eumelus/author/Ziv:B) B, [Saaroni](http://www.citeulike.org/user/eumelus/author/Saaroni:H) H, [Alpert](http://www.citeulike.org/user/eumelus/author/Alpert:P) P (2004) [The factors governing the summer regime of the eastern Mediterranean](http://www.citeulike.org/user/eumelus/article/6463629). Int J Climatol 24: 1859-1871

**Table S1**  Modeled effects of climate change on water resources for 1961-1990, 2040-2069 and 2070-2099. RR is mean precipitation rate, IWR are mean internal renewable water resources. ΔRR represents changes relative to 1961-1990. Decreases are in red

| Country | RR  1961-90  (mm/yr) | RR  2040-69  (mm/yr) | RR  2070-99  (mm/yr) | ΔRR  2040-69  (%) | IWR  2040-69  (km^3^/yr) | ΔRR  2070-99  (%) | IWR  2070-99  (km^3^/yr) |
| --- | --- | --- | --- | --- | --- | --- | --- |
| Albania | 1452 | 1252 | 1249 | -13.8 | 23.2 | -14.0 | 23.1 |
| Bahrain | 98 | 103 | 142 | 5.2 | 0.0 | 45.0 | 0.0 |
| Bulgaria | 755 | 688 | 661 | -8.8 | 19.1 | -12.5 | 18.4 |
| Cyprus | 330 | 263 | 274 | -20.3 | 0.6 | -17.0 | 0.6 |
| FYROM | 814 | 734 | 701 | -9.8 | 4.9 | -13.9 | 4.6 |
| Greece | 730 | 601 | 567 | -17.7 | 47.7 | -22.4 | 45.0 |
| Iraq | 163 | 158 | 155 | -3.0 | 34.1 | -4.9 | 33.5 |
| Israel | 174 | 151 | 141 | -12.9 | 0.7 | -18.8 | 0.6 |
| Jordan | 88 | 73 | 69 | -16.6 | 0.6 | -21.1 | 0.5 |
| Kuwait | 63 | 82 | 83 | 30.7 | 0.0 | 31.4 | 0.0 |
| Lebanon | 649 | 494 | 449 | -23.9 | 3.7 | -30.9 | 3.3 |
| Palestinian terr. | 201 | 172 | 154 | -14.7 | 0.7 | -23.3 | 0.6 |
| Qatar | 76 | 97 | 118 | 27.9 | 0.1 | 55.2 | 0.1 |
| Syria | 219 | 189 | 184 | -13.7 | 6.0 | -16.1 | 5.9 |
| Turkey | 791 | 704 | 694 | -11.0 | 202.0 | -12.2 | 199.2 |

**
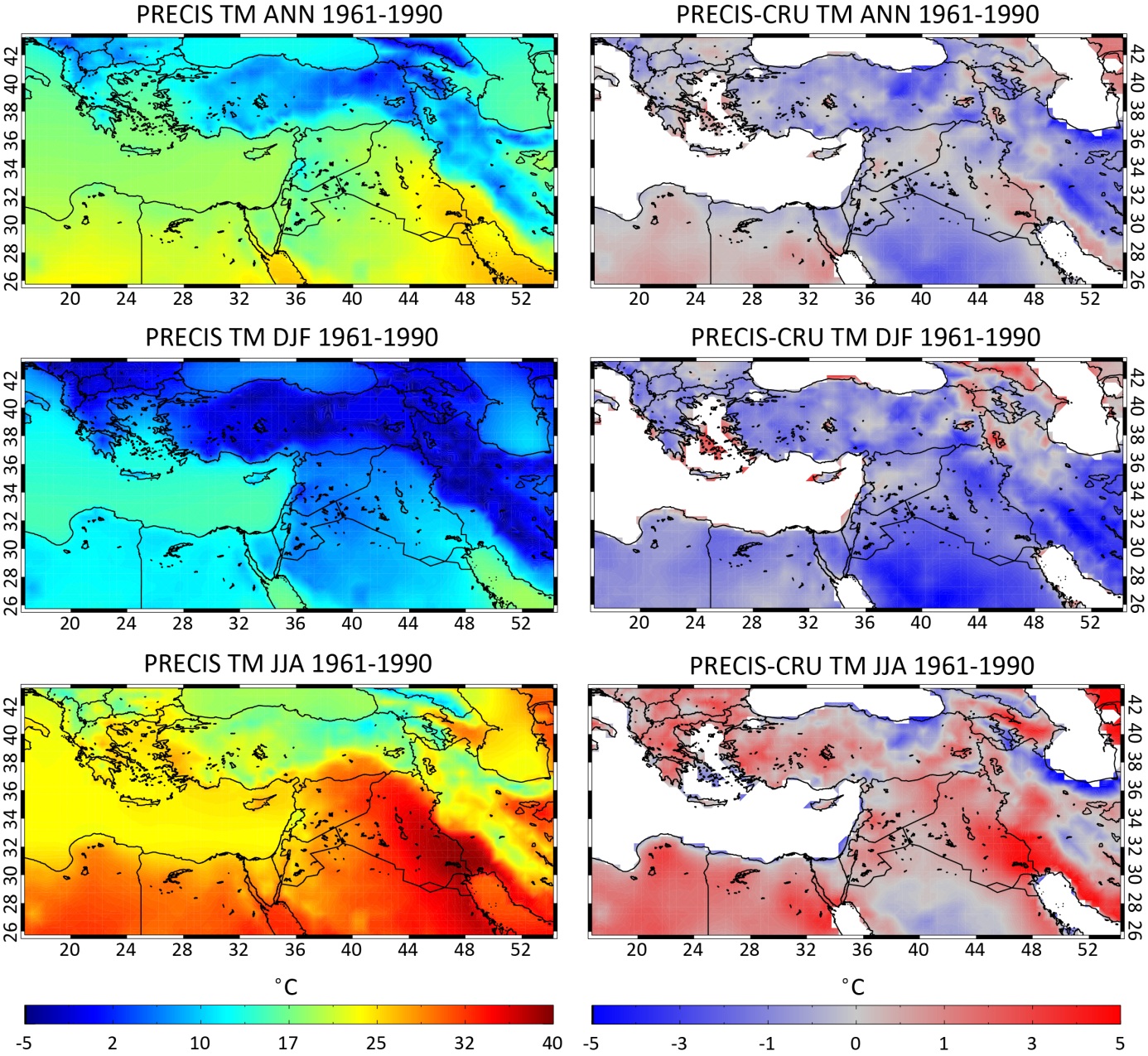
**

**Fig. S1** Patterns of annual (top), winter (DJF, middle) and summer (JJA, bottom) mean temperature (TM). Left panels show PRECIS output mapped on the CRU grid, and right panels show the differences between PRECIS and CRU

**
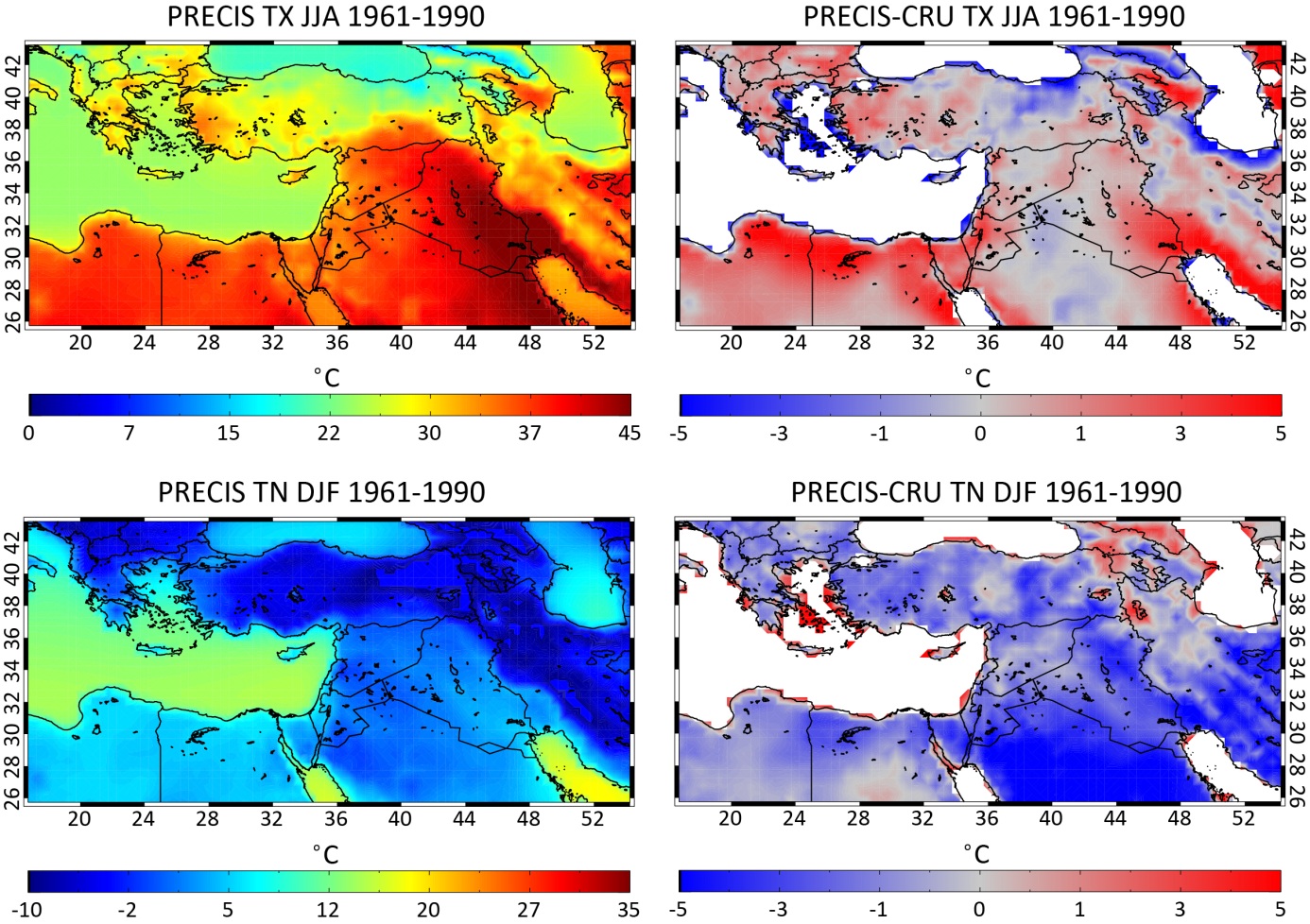
**

**Fig. S2** Patterns of the mean summer maximum (JJA, top) and mean winter minimum (DJF, bottom) temperatures, TX and TN, respectively. Left panels show PRECIS output mapped on the CRU grid, and right panels show the differences between PRECIS and CRU

**
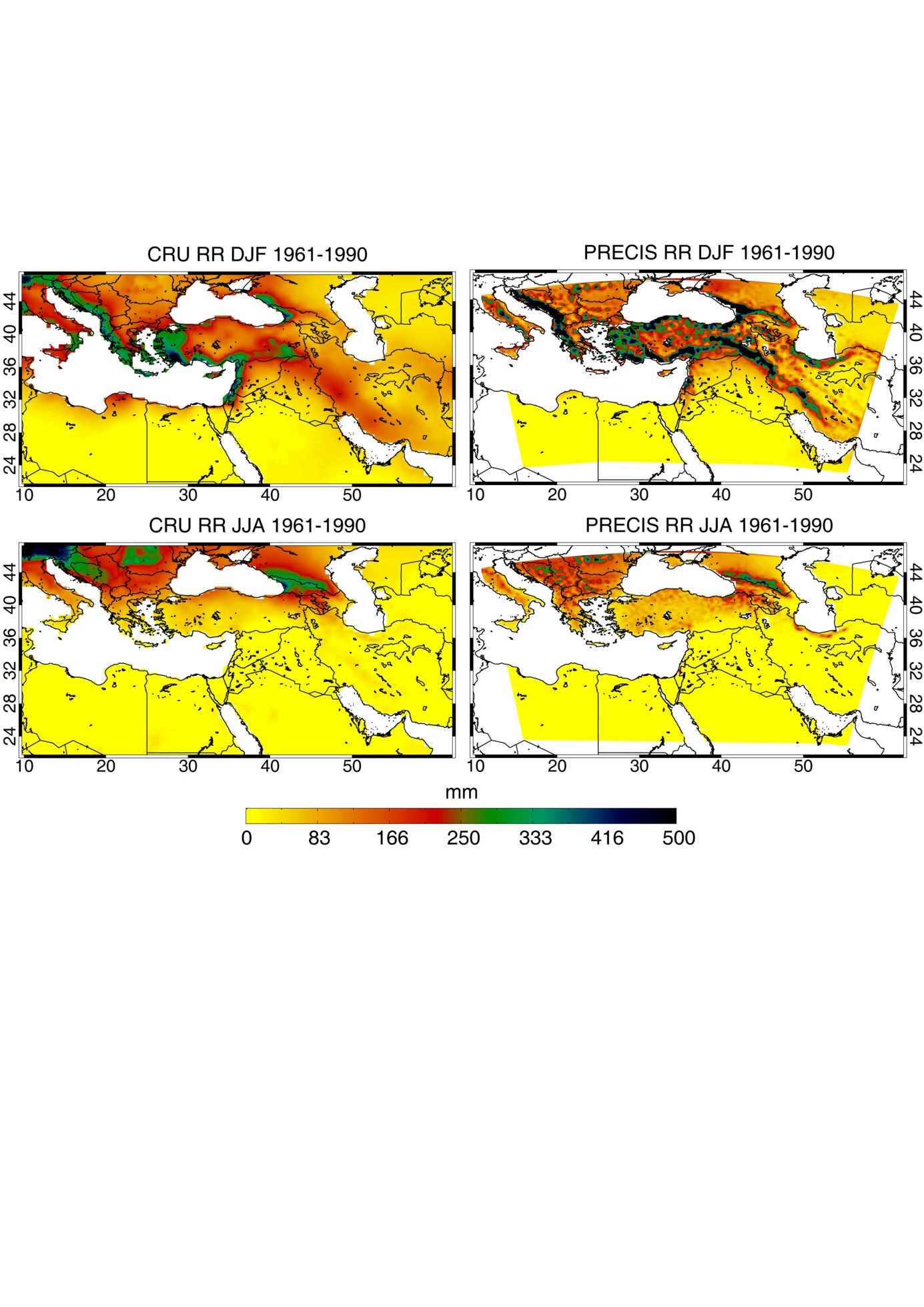
**

**Fig. S3** Patterns of mean winter (DJF, top) and summer (JJA, bottom) rainfall rates (RR). Left panels are based on CRU data and right panels on PRECIS output

**
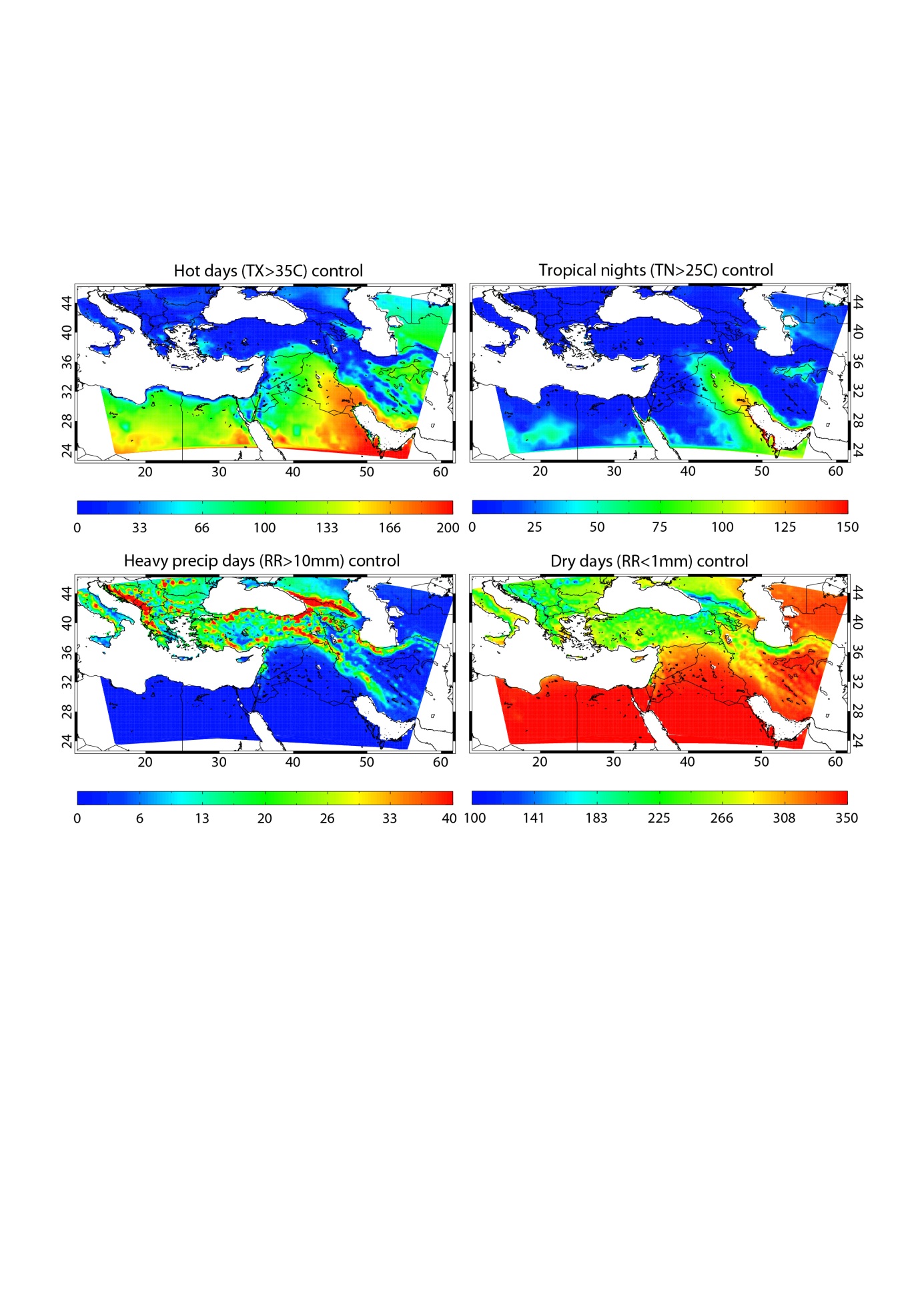
**

**Fig. S4** Patterns of temperature (TX, TN) and precipitation (RR) indices, calculated from PRECIS output, showing the mean number of days per year during the control period (1961-1990)


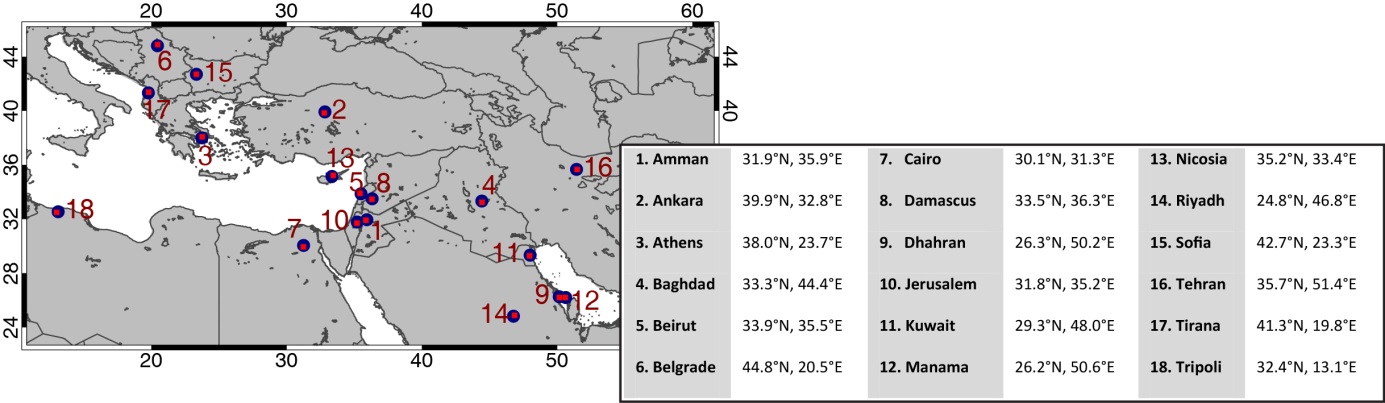


**Fig. S5** Selected locations, mostly capitals, in the CRU (blue) and PRECIS (red) datasets for which long-term temperature trends are calculated (Fig. 4)

**
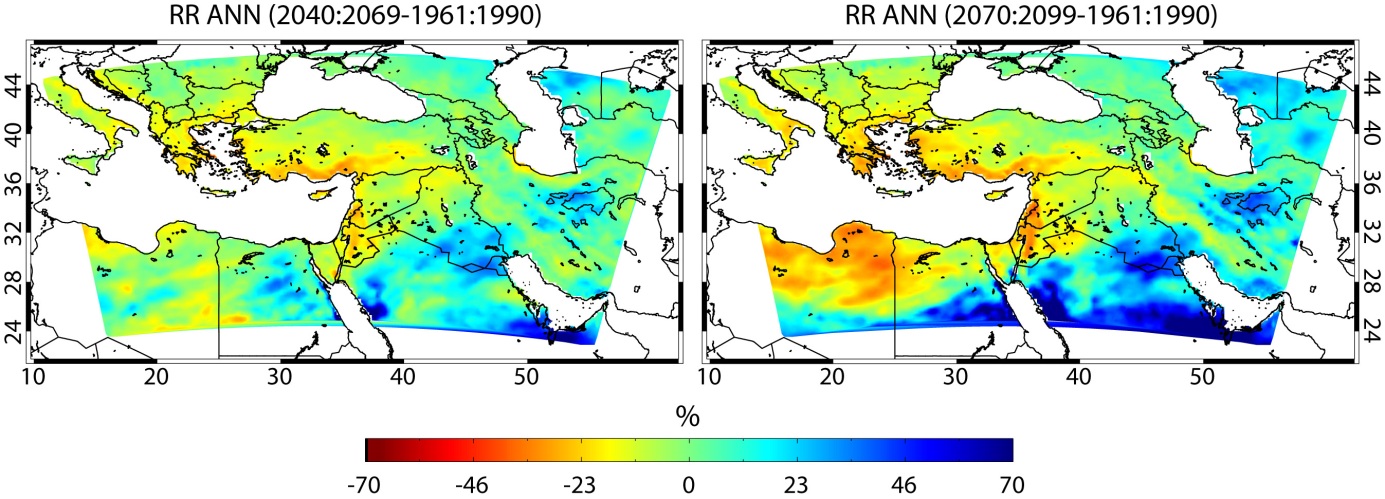
Fig. S6** Patterns of changing annual mean precipitation (RR) in percent, calculated from PRECIS output. The left panel shows the mean changes for 2040-2069 and the right panel for 2070-2099 relative to the control period 1961-1990

**
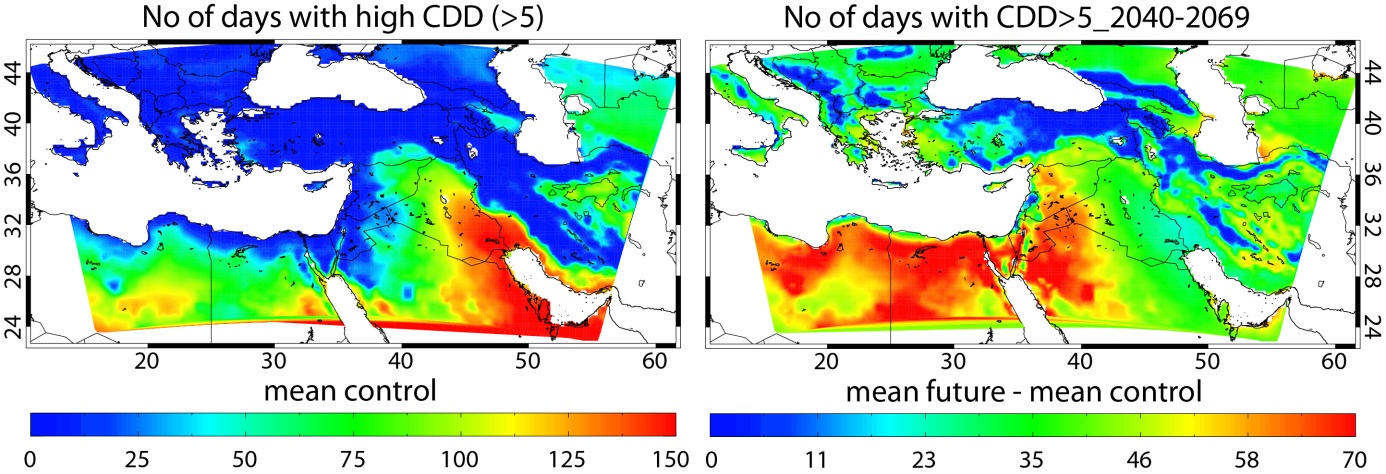
**

**Fig. S7** Patterns of mean number of heavy cooling degree days/year (CDD>5°C) for the control period 1961-1990 (left) and additional CDD>5°C days for the period 2040-2069, calculated from PRECIS output


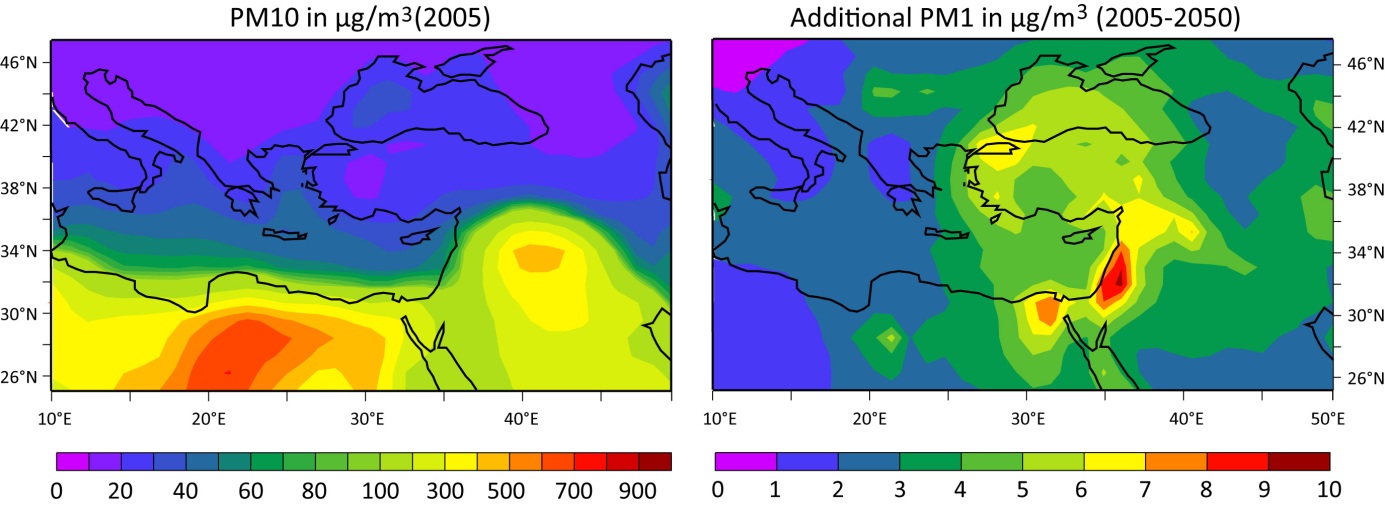


**Fig. S8** Annual average PM10 in 2005 (left). Right: increasing anthropogenic aerosols (PM1) in 2050, calculated by applying an intermediate air pollution increase scenario (equivalent to A1B for greenhouse gases). Note that PM10 is mostly natural (e.g. dust) of which the 2005 and 2050 emissions have been kept identical


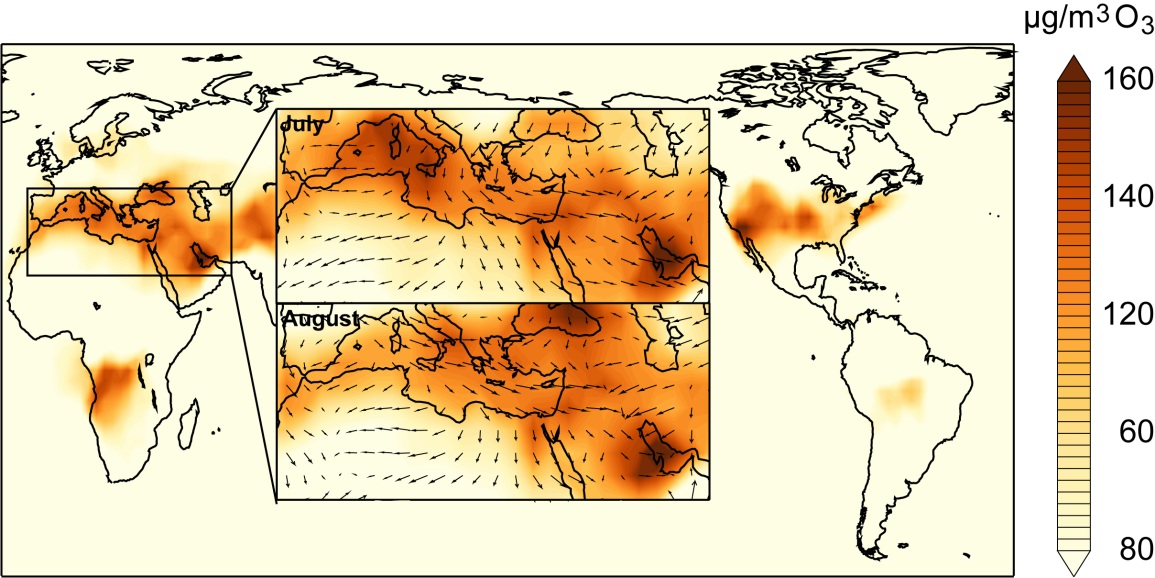


**Fig. S9** Model calculated ozone concentrations near the surface in summer 2006, indicating that air quality standards for ozone are strongly exceeded. The arrows in the inserts represent the near-surface winds, indicating the directions of air pollution transports
